# Synthesis and Characterization of Potential Dimers of Gatifloxacin – an Antibacterial Drug

**DOI:** 10.3797/scipharm.1212-21

**Published:** 2013-02-18

**Authors:** Srinivas Garaga, Ambati V. Raghava Reddy, Koilpillai Joseph Prabahar, Raghu Babu Korupolu, Paul Douglas Sanasi

**Affiliations:** 1 Chemical Research and Development Department, Aurobindo Pharma Ltd, Survey No: 71&72, Indrakaran (V), Sangareddy (M), Medak Dist., Hyderabad – 502 329, Andhra Pradesh, India.; 2 Department of Engineering Chemistry, A. U. College of Engineering (A), Andhra University, Visakhapatnam – 530 003, Andhra Pradesh, India.

**Keywords:** Gatifloxacin, Piperazine dimers, Synthesis, Characterization

## Abstract

Gatifloxacin is an antibacterial agent belonging to the fourth-generation fluoroquinolone family. Four piperazine-linked fluoroquinolone dimers of Gatifloxacin were observed during the laboratory process for Gatifloxacin and they were identified. The present work describes the origin, synthesis, characterization, and control of these dimers along with the synthesis of Despropylene Gatifloxacin (metabolite).

## Introduction

Gatifloxacin **1** is chemically known as 1-cyclopropyl-6-fluoro-8-methoxy-7-(3-methylpiperazin-1-yl)-4-oxo-1,4-dihydroquinoline-3-carboxylic acid, one of the most important broad-spectrum antibacterial agents and a member of the fourth-generation fluoroquinolone family. Gatifloxacin **1** is marketed by Bristol-Myers Squibb in the brand name of Tequin^®^ for the treatment of respiratory tract infections. It is available as aqueous solutions for intravenous therapy. The ophthalmic solution is marketed by Allergan in the brand name of Zymer^®^.

The presence of impurities in an Active Pharmaceutical Ingredient (API) will influence the quality and safety of the drug product. In the regulatory guidelines of the International Conference on Harmonization (ICH), it is recommended that impurities more than 0.1% [1] should be identified and characterized. Impurities are required in pure form to check the analytical performance characteristics such as specificity, linearity, range, accuracy, precision, limit of detection (LOD), limit of quantification (LOQ), robustness, system suitability testing, and relative retention factor [2].

During the process development of Gatifloxacin **1** in the laboratory, we observed four unknown impurities along with the known impurities [3, 4]. These unknown impurities were detected, monitored, and their structures were tentatively assigned on the basis of their fragmentation patterns in LC-MS. In the present work, the identified impurities of Gatifloxacin were synthesized and characterized by various spectroscopic techniques and further confirmed by co-injection studies in the qualitative HPLC analysis.

## Results and Discussion

Gatifloxacin (**1**) has been synthesized by known literature methods [5–10]. Our route of synthesis of Gatifloxacin is shown in Scheme 1. 1-Cyclopropyl-6,7-difluoro-8-methoxy-4-oxo-1,4-dihydroquinoline-3-carboxylic acid ethyl ester **2** was treated with boron trifluoride diethyl etherate in methyl isobutyl ketone to give the corrsponding borondifluoride chelate **3**. Condensation of **3** with 2-methylpiperazine yielded 1-cyclopropyl-6-fluoro-1,4-dihydro-8-methoxy-7-(3-methyl-1-piperazinyl)-4-oxo-3-quinolinecarboxylic acid borondifluoride chelate (Gatifloxacin borondifluoride chelate) **4**. Hydrolysis of **4** with triethylamine yielded 1-cyclopropyl-6-fluoro-1,4-dihydro-8-methoxy-7-(3-methyl-1-piperazinyl)-4-oxo-3-quinoline-carboxylic acid (Gatifloxacin) **1**. To the best of our knowledge, a detailed impurity profile study is not yet cited anywhere, except for the metabolite **14**.

The impurities of Gatifloxacin dimers 1–4 and a metabolite **14**, which were identified in the synthetic process of Gatifloxacin **1** are given in Table 1. Origin, synthesis, and structural characterization of each of the impurities are presented below individually. Each of these synthesized impurities were characterized by conventional spectroscopic studies and the presence of these synthetically prepared dimers in the Gatifloxacin sample was confirmed by spiking the dimer samples individually with the Gatifloxacin sample and analyzed by the qualitative HPLC method. These studies confirmed the formation of dimer impurities **1–4** during the manufacturing process of Gatifloxacin **1**.

### Gatifloxacin dimer-1

Gatifloxacin dimer-1 **6** originated from the substitution reaction of the piperazine moiety of Gatifloxacin **1** with the fluoro group present at the C-7 position of 8-methoxyquinoline carboxylic acid **7** during the hydrolysis of Gatifloxacin borondifluoride chelate **4**.

Compound **6** was independently prepared by the condensation of compound **1** with compound **3** in DMSO to produce compound **5**, which was hydrolyzed with triethylamine in methanol to produce compound **6** (Scheme 2). The mass spectrum showed a molecular ion at m/z 651.1 amu [(M+H)^+^] and a sodium adduct at m/z 673.1 amu [(M+Na)^+^]. In comparison with Gatifloxacin **1**, twice the number of quinoline moiety protons in ^1^H NMR were observed.

### Gatifloxacin dimer-2

Gatifloxacin dimer-2 **9** originated from the condensation of the piperazine moiety of Gatifloxacin **1** with the carboxylic acid of 8-methoxyquinolinecarboxylic acid **7** during the hydrolysis of Gatifloxacin borondifluoride chelate **4**.

Compound **8** was independently prepared from compound **7.** Reaction of **7** with thionyl chloride in dichloromethane to produce compound **8**, which was further reacted with Gatifloxacin **1** in dichloromethane in presence of diisopropylethylamine, produced compound **9** (Scheme 3). The mass spectrum showed a molecular ion at m/z 653.2 amu [(M+H)^+^]. The amide group was confirmed by observing C=O stretching at 1599 cm^−1^ in the IR spectrum. In comparison with Gatifloxacin **1**, twice the number of quinoline moiety protons in ^1^H NMR and twice the number of carbon signals in ^13^C NMR were observed.

### Gatifloxacin dimer-3

The Gatifloxacin dimer-3 **11** (~0.07%) impurity was formed by the self-condensation of Gatifloxacin **1** during the hydrolysis of Gatifloxacin borondifluoride chelate **4**.

Compound **11** was independently prepared from compound **1**. Reaction of **1** with thionyl chloride in dichloromethane to produce compound **10**, which was further reacted with compound **1** in dichloromethane in presence of diisopropylethylamine, produced compound **11** (Scheme 4). The mass spectrum showed a molecular ion at m/z 733.5 amu [(M+H)^+^]. The amide group was confirmed by observing C=O stretching at 1582 cm^−1^ in the IR spectrum. In comparison with Gatifloxacin **1**, twice the protons of the Gatifloxacin **1** moiety in ^1^H NMR, and twice the number of carbon signals in ^13^C NMR were observed.

### Despropylene Gatifloxacin

Despropylene Gatifloxacin **14** is a known metabolite of Gatifloxacin **1**[11–14] and it originated due to the presence of ethylenediamine in 2-methylpiperazine raw material, which condensed with compound **3** to give compound **14**. It also originated by the photolytic degradation of Gatifloxacin [12].

Compound **14** was independently prepared from compound **3** and **12** in acetonitrile to produce compound **13**. Compound **13** was treated with triethylamine in methanol to produce compound **14** (Scheme 5). The mass spectrum showed a molecular ion at m/z 336.1362 amu [(M+H)^+^]. The structure of compound **14** was further proven by the ^1^H NMR spectrum showing CH_2_ protons at δ 3.12 (2H) & 3.75 (2H) ppm and also NH_2_ protons at δ 7.89 (3H) ppm.

### Gatifloxacin dimer-4

Gatifloxacin dimer-4 **16** originated by the condensation of ethylenediamine, present as an impurity in the raw material, and 2-methylpiperazine with two molecules of compound **3**.

Compound **16** was independently prepared from compound **14** from the reaction with **3** in DMSO to produce compound **15**, which was further reacted with triethylamine in methanol to produce Gatifloxacin dimer-4 **16** (Scheme 6). The mass spectrum showed a molecular ion at m/z 611.2 amu [(M+H)^+^] and its sodium adduct at m/z 633.1 amu [(M+Na)^+^]. In comparison with compound **14**, we observed twice the number of quinoline moiety protons in the ^1^H NMR.

Gatifloxacin dimers 1–4, **6, 9, 11, 16** are novel and process-related compounds. These impurities were eliminated during the purification of Gatifloxacin **1**. Compounds **14 & 16** originated due to the presence of the ethylenediamine impurity found in the raw material 2-methylpiperazine, and thus ethylenediamine is controlled within a specified limit (0.1%) in the specifications of the 2-methylpiperazine raw material.

## Experimental

Solvents and reagents were obtained from the commercial sources and used without purification.^1^H NMR and ^13^C NMR spectral data were performed on the Bruker-Avance 300-MHz spectrometer in DMSO-d_6_. The chemical shift values were reported on the δ scale in parts per million (ppm), downfield from tetramethylsilane (TMS) as an internal standard. IR spectra were recorded in the solid state as KBr pellets using a Perkin-Elmer FT-IR spectrophotometer. The mass spectrum was recorded using a Perkin-Elmer PE SCIEX-API 2000, equipped with ESI source used online with a HPLC system after the ultraviolet (UV) detector.

### Gatifloxacin dimer-1 (7,7′-(2-Methylpiperazine-1,4-diyl)bis(1-cyclopropyl-6-fluoro-8-methoxy-4-oxo-1,4-dihydroquinoline-3-carboxylic acid, 6)

To a suspension of 1-cyclopropyl-6,7-difluoro-8-methoxy-4-oxo-1,4-dihydroquinoline-3-carboxylic acid borondifluoride chelate **3** (25 g, 7.28 mmol) in DMSO (275 mL), 1-cyclopropyl-6-fluoro-1,4-dihydro-8-methoxy-7-(3-methylpiperazin-1-yl)-4-oxo-quinoline-3-carboxylic acid **1** (27.3 g, 7.28 mmol) was added at room temperature. The reaction mass was heated to 80–85 °C and stirred for 24 h at 80–85 °C. The reaction mass was cooled to room temperature and added triethylamine (7.36 g, 7.28 mmol). Further, the reaction mass was heated and stirred for another 10 h at 80–85 °C. The reaction mass was cooled to 5–10 °C, filtered the product, and dried to obtain compound **6**, which was purified by crystallizing from methanol to yield a white solid **6** (8 g, 12.7% yield); HPLC Purity: 82.19%; IR (KBr pellet, cm^−1^): 3442 (OH, Str.), 3082 (Aromatic C-H, Str.), 2942, 2867 (Aliphatic C-H, Str.), 1727, 1619 (C=O, Str.), 1444 (-CH_2_, bend.), 777, 739 (Aromatic CH out-of-plane bend); ^1^H-NMR (D_2_O+1drop of NaOD, 300 MHz): 0.8–1.07 (m, 8H, cyclopropyl), 1.092 (m, 2H, cyclopropyl), 1.132 (m, 3H, CH_3_), 3.74–3.84 (S, 6H, OCH_3_), 3.1 (m, 1H, piperazine), 3.3–3.46 (m, 6H, piperazine), 7.66–7.693 (m, 2H), 8.49–8.55 (m, 2H), MS *m/z*: 651.2271 [(M+H)^+^].

### Gatifloxacin dimer-2 (1-Cyclopropyl-7-{4-[(1-cyclopropyl-6,7-difluoro-8-methoxy-4-oxo-1,4-dihydroquinolin-3-yl)carbonyl]-3-methylpiperazin-1-yl}-6-fluoro-8-methoxy-4-oxo-1,4-dihydroquinoline-3-carboxylic acid, 9)

To a suspension of 1-cyclopropyl-6,7-difluoro-8-methoxy-4-oxo-1,4-dihydroquinoline-3-carboxylic acid **7** (10 g, 3.38 mmol) in dichloromethane (100 mL), thionyl chloride (6.05 g, 5.08 mmol) was added at room temperature. The reaction mass was heated to 35–40 °C and stirred for 2 h to complete the formation of 1-cyclopropyl-6,7-difluoro-8-methoxy-4-oxo-1,4-dihydroquinoline-3-carbonyl chloride **8**. The reaction mass was concentrated under reduced pressure to remove excess thionyl chloride and dissolved the residue in dichloromethane (100 mL). In a separate flask, 1-cyclopropyl-6-fluoro-1,4-dihydro-8-methoxy-7-(3-methylpiperazin-1-yl)-4-oxo-quinoline-3-carboxylic acid **1** (12.71 g, 3.38 mmol) was dissolved in dichloromethane (750 mL) and added N,N-diisopropylethylamine (8.5 g, 6.79 mmol) at room temperature. Thereafter, the above prepared acid chloride **8** solution was added at room temperature. After 1 h stirring at 20–25 °C, the reaction mass was washed with DM water (250 mL). DM water (250 mL) was added to the organic layer and adjusted the pH to 5.5 with 10% aqueous hydrochloric acid. The organic layer was taken and concentrated at >40 °C under vacuum to obtain a residue, which was triturated with ethyl acetate to obtain a white solid **9**. (17.5 g, 80% yield); HPLC Purity: 91.71%; IR (KBr pellet, cm^−1^): 3452 (OH, Str.), 3050 (Aromatic C-H, Str.), 2950, 2850 (Aliphatic C-H, Str.), 1731,1621,1599 (C=O, Str.), 1469 (-CH_2_, bend.), 783, 760 (Aromatic CH out-of-plane bend); ^1^H-NMR (DMSO-D_6_, 300 MHz): 1.03–1.14 (m, 8H, cyclopropyl), 1.34 (s, 2H, cyclopropyl), 1.35 (s, 3H, CH_3_), 3.73 (s, 3H, OCH_3_), 3.42 (s, 3H, OCH_3_), 3.44–3.49 (s, 3H, piperazine), 4.05–4.08 (s, 4H, piperazine), 7.75–7.87 (s, 2H), 8.19–8.71 (s, 2H); ^13^C-NMR (DMSO-D_6_): 8.9, 15.36, 16.21, 37.01, 40.6, 42.5, 45.06, 50.61, 54.73, 55.24, 63.14, 63.67, 106.47, 106.94, 117.12, 121.15, 123.68, 132.65, 134.18, 139.8, 140.69, 146.58, 148.83, 149.05, 150.62, 154.28, 156.77, 164.66, 165.25, 170.84, 176.3. MS *m/z*: 653.2220 [(M+H)^+^].

### Gatifloxacin dimer-3 (1-Cyclopropyl-7-(4-{[1-cyclopropyl-7-fluoro-8-methoxy-6-(3-methylpiperazin-1-yl)-4-oxo-1,4-dihydroquinolin-3-yl]carbonyl}-3-methylpiperazin-1-yl)-6-fluoro-8-methoxy-4-oxo-1,4-dihydroquinoline-3-carboxylic acid, 11)

To a suspension of 1-cyclopropyl-6-fluoro-1,4-dihydro-8-methoxy-7-(3-methyl-1-piperazin-yl)-4-oxo-3-quinolinecarboxylic acid **1** (10 g, 2.66 mmol) in dichloromethane (100 mL), thionyl chloride (9.52 g, 8 mmol) was added at room temperature. The reaction mass was heated to 35–40 °C and stirred for 2 h to complete the formation of 1-cyclopropyl-6-fluoro-1,4-dihydro-8-methoxy-7-(3-methyl-1-piperazinyl)-4-oxo-3-quinolinecarbonyl chloride **10**. The reaction mass was concentrated under reduced pressure to remove excess thionyl chloride and dissolved the residue in dichloromethane (100mL). In a separate flask, 1-cyclopropyl-6-fluoro-1,4-dihydro-8-methoxy-7-(3-methylpiperazin-1-yl)-4-oxoquinoline-3-carboxylic acid **1** (10 g, 2.66 mmol) was dissolved in dichloromethane (750 mL), N,N-diisopropylethylamine (13.4 g, 10.66 mmol) was added and cooled the reaction mass to 0–5 °C. Thereafter, the above prepared acid chloride **10** solution was added at 0–5 °C. After 1 h stirring at 20–25 °C, the reaction mass was washed with DM water (250 mL). DM water (250 mL) was added to the organic layer and adjusted pH to 7.0 with 10% aqueous hydrochloric acid. The organic layer was separated, washed with DM water (250 mL), and concentrated at 35–40 °C under reduced pressure to obtain a residue, which was triturated with hexanes and further purified by using column chromatography to obtain a white solid **11**. (12.2 g, 62.6% yield); HPLC Purity: 99.42%; IR (KBr pellet, cm^−1^): 3425 (OH, Str.), 3084 (Aromatic C-H, Str.), 2978, 2847 (Aliphatic C-H, Str.), 1725, 1620, 1589 (C=O, Str.), 1448 (-CH_2_, bend.), 734, 806 (Aromatic CH out-of-plane bend); ^1^H-NMR (DMSO-D_6_, 300 MHz): 0.91–1.1 (m, 8H, cyclopropyl), 1.1–1.33 (m, 6H, CH_3_), 1.9 (s, 2H, cyclopropyl), 3.74 (s, 6H, OCH_3_), 2.7–3.35 (m, 12H, piperazine), 3.76 (s, 1H, piperazine), 3.97 (s, 1H, piperazine), 7.59–7.63 (d, 1H), 7.76–7.8 (s, 1H), 8.1 (s, 1H), 8.71 (s,1H); ^13^C-NMR (DMSO-D_6_): 8.9, 19.08, 21.29, 40.67, 45.93, 48.6, 50.73, 57.87, 62.53, 63.68, 106.72, 121.17, 122.65, 133.57, 134.21, 137.87, 139.84, 145.86, 146.15, 150.65, 153.86, 154.29, 156.32, 156.78, 165.12, 165.68, 172.21, 176.31; MS *m/z*: 733.3148 [(M+H)^+^]

### Despropylene Gatifloxacin (7-[(2-Aminoethyl)amino]-1-cyclopropyl-6-fluoro-8-methoxy-4-oxo-1,4-dihydroquinoline-3-carboxylic acid, 14)

To a suspension of 1-cyclopropyl-6,7-difluoro-8-methoxy-4-oxo-1,4-dihydroquinoline-3-carboxylic acid borondifluoride chelate 3 (25 g, 7.28 mmol) in acetonitrile (200 mL), ethylenediamine 12 (8.75 g, 14.58 mmol) was added at room temperature. The reaction mass was stirred for 16 h at 25–30 °C. The resulting product **13** was cooled to 5–10 °C, filtered, and suspended in methanol (232 mL) at 25–30 °C. Triethylamine (7.65 g, 7.57 mmol) was added to the above at 25–30 °C and heated the slurry mass to 60–65 °C for 16 h. Cooled the slurry mass to 25–30 °C, filtered, and dried to obtain a white solid **14**. (21 g, 74.3% yield); HPLC Purity: 99.64%; IR (KBr pellet, cm^−1^): 3332(OH, Str.), 3083 (Aromatic C-H, Str.), 2967 (Aliphatic C-H, Str.), 1620 (C=O, Str.), 1466 (-CH_2_, bend.), 770.8, 747 (Aromatic CH out-of-plane bend); ^1^H-NMR (DMSO-D_6_+ TFA, 300 MHz): 1.04–1.17 (d,.4H, cyclopropyl), 3.12 (t, 2H, CH_2_), 3.75 (s, 5H, CH_2_, OCH_3_), 4.18 (s, 1H), 7.74–7.79 (d, 1H), 7.89 (s, 3H), 8.68 (s, 1H); ^13^C-NMR (DMSO-D_6_): 9.07, 44.62, 61.39, 105.93, 106.84, 107, 115.28, 133.37, 137.23, 149.17, 149.95, 151.61, 165.95, 176; MS *m/z*: 336.1362 [(M+H)^+^]

### Gatifloxacin dimer-4 (7,7′-(Ethane-1,2-diyldiimino)bis(1-cyclopropyl-6-fluoro-8-methoxy-4-oxo-1,4-dihydroquinoline-3-carboxylic acid, 16)

To a suspension of 7-[(2-aminoethyl)amino]-1-cyclopropyl-6-fluoro-8-methoxy-4-oxo-1,4-dihydroquinoline-3-carboxylic acid **14** (5 g, 1.49 mmol) in DMSO (40 mL), 1-cyclo propyl-6,7-difluoro-8-methoxy-4-oxo-1,4-dihydroquinoline-3-carboxylic acid borondifluoride chelate **3** (5.11 g, 1.49 mmol) was added. The reaction mass was heated to 70–75 °C and maintained at the same temperature for 16 h. Thereafter, the resulting slurry mass was cooled to 25–30 °C and filtered the product **14**. This wet material was suspended in the mixture of methanol (70 mL) and sodium hydroxide (0.36 g, 0.9 mmol). The reaction mass was heated to 40–45 °C and stirred for 4 h at 40–45 °C. The reaction mass was cooled to 25–30 °C. DM water (200 mL) was added to the slurry mass and adjusted pH to 7.0 with 10% aqueous hydrochloric acid at 25–30 °C, the resulting product was filtered and dried to obtain a white solid **16** (4.5 g, 50% yield); HPLC Purity: 99.45%; IR (KBr pellet, cm^−1^): 3366 (OH, Str.), 3077 (Aromatic C-H, Str.), 2964 (Aliphatic C-H, Str.), 1725, 1620 (C=O, Str.), 1465 (-CH_2_, bend.), 771, 749 (Aromatic CH out-of-plane bend); ^1^H-NMR (DMSO-D_6_+ TFA, 300 MHz): 1.02–1.13 (m,.8H, cyclopropyl), 3.68 (s, 6H, OCH_3_), 3.76 (s, 4H, CH_2_), 4.14 (m, 2H), 7.67–7.72 (d, 2H), 8.64 (s, 2H); MS *m/z*: 611.1953 [(M+H)^+^].

## Supporting Information

Supporting information containing ^1^H-NMR spectra (**6**, **9**, **11**, **14**, **16**), ^13^C-NMR spectra (**9**, **11**, **14**), HRMS & elemental analyses (**6**, **9**, **11**, **14**, **16**), IR spectra (**6**, **9**, **11**, **14**, **16**), HPLC purity spectra (**6**, **9**, **11**, **14**, **16**), and the impurity mixture chromatogram of dimers are available in the online version (Format: PDF, Size: ca. 1.0 MB): http://dx.doi.org/10.3797/scipharm.1212-21.

## Figures and Tables

**Sch. 1:**
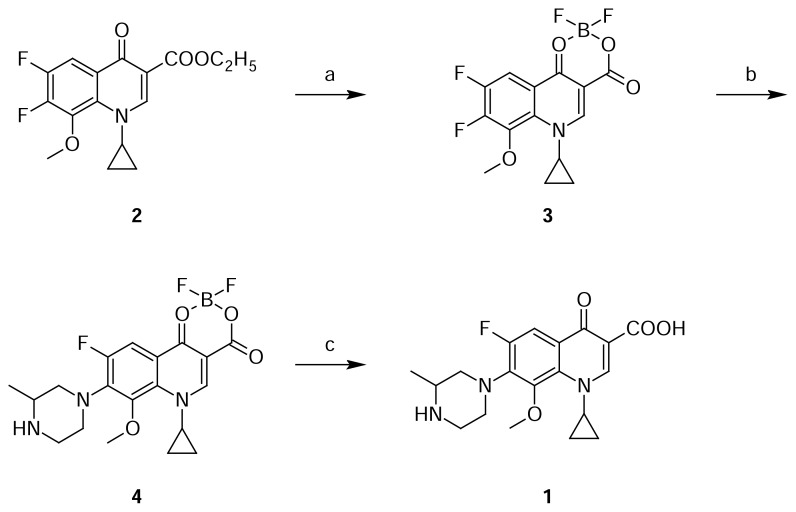
Reported synthetic scheme of gatifloxacin **1**. Reagents and conditions: (a) borontrifluoride diethyletherate, MIBK; yield: 85%; (b) 2-methylpiperazine, acetonitrile; yield: 85%; (c) TEA, methanol; yield: 90%.

**Sch. 2:**
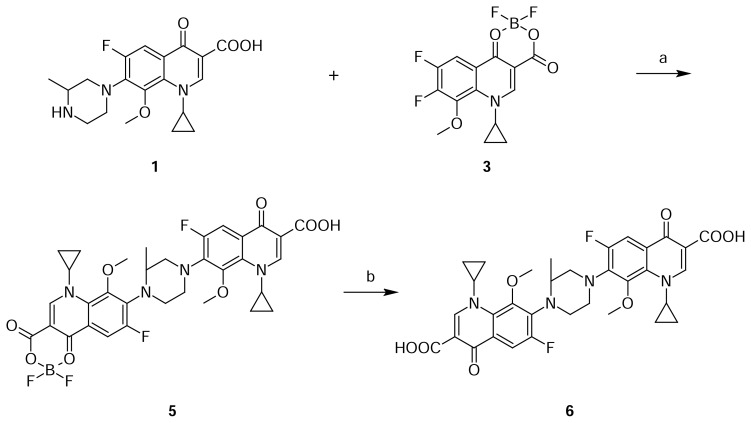
Synthetic scheme of Gatifloxacin dimer-1 **6**. Reagents and conditions: (a) DMSO; (b) TEA, methanol; Overall yield: 12.7%.

**Sch. 3:**
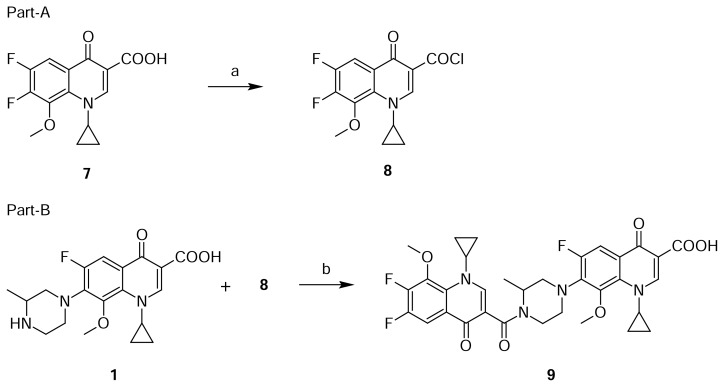
Synthetic scheme of Gatifloxacin dimer-2 **9**. Reagents and conditions: (a) SOCl_2_, DCM; (b) DIPEA, DCM; Overall yield: 80%.

**Sch. 4:**
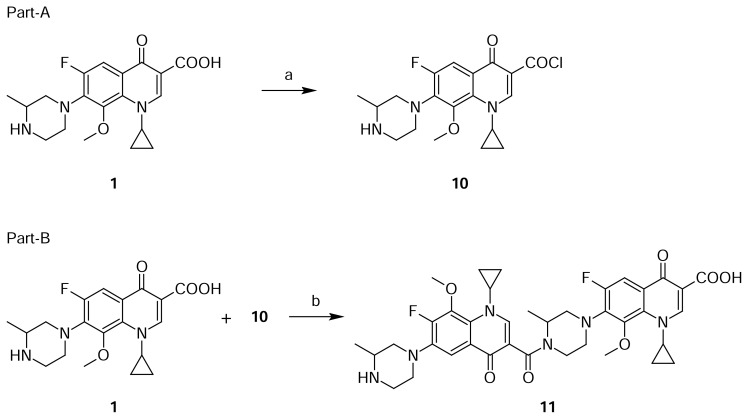
Synthetic scheme of Gatifloxacin dimer-3 **11**. Reagents and conditions: (a) SOCl_2_, DCM; (b) DIPEA, DCM: Overall yield: 62.6%.

**Sch. 5:**
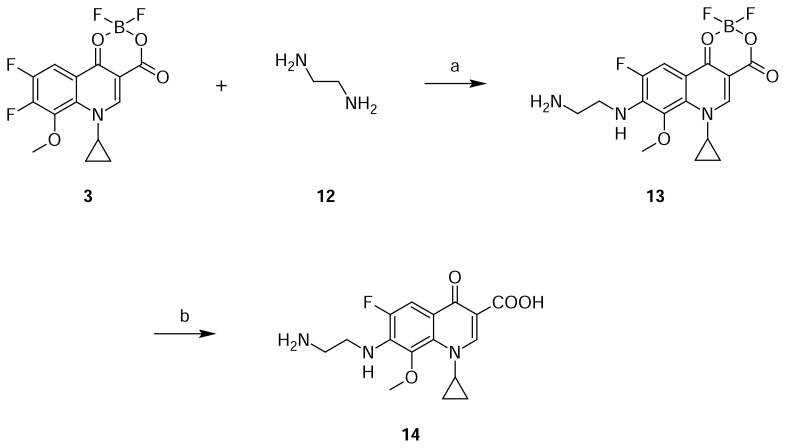
Synthetic scheme of Despropylene Gatifloxacin **14**. Reagents and conditions: (a) acetonitrile; (b) TEA, methanol; Overall yield: 74.3%.

**Sch. 6:**
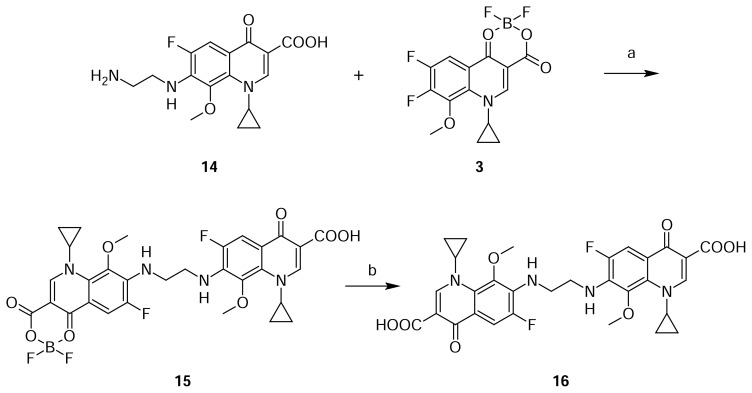
Synthetic scheme of Gatifloxacin dimer-4 **16**. Reagents and conditions: (a) DMSO; (b) TEA, methanol; Overall yield: 50%.

**Tab. 1:** Impurities of Gatifloxacin, their origin and syntheses

Impurity (Cpd. Nr.)	Origin	Synthesis
Gatifloxacin dimer-1 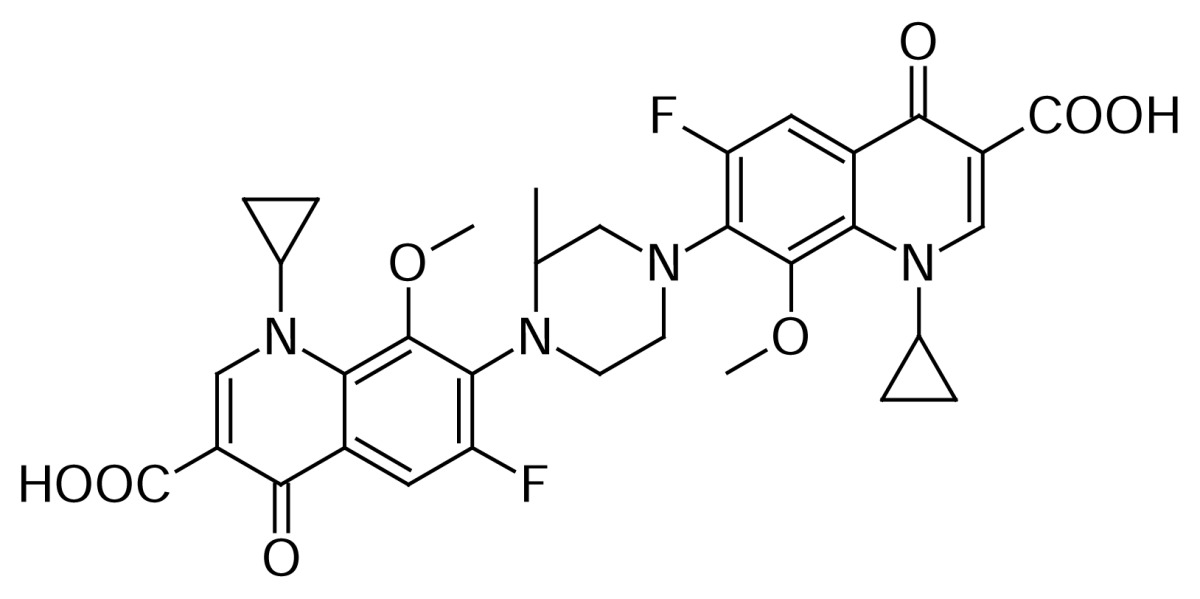 **6**	Condensation of **1** and **3**	Scheme 2
Gatifloxacin dimer-2 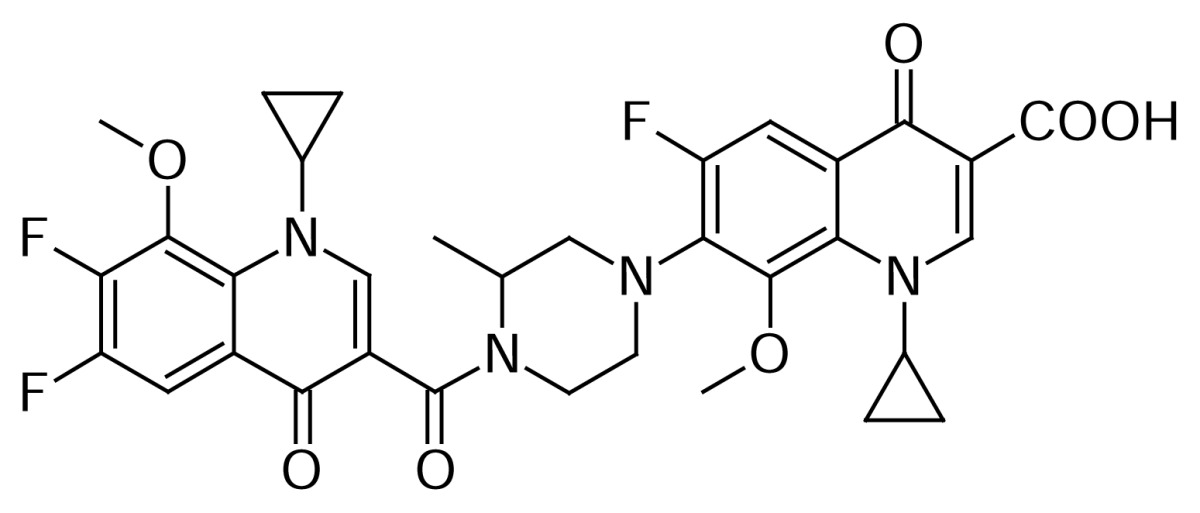 **9**	Condensation of **1** and **7**	Scheme 3
Gatifloxacin dimer-3 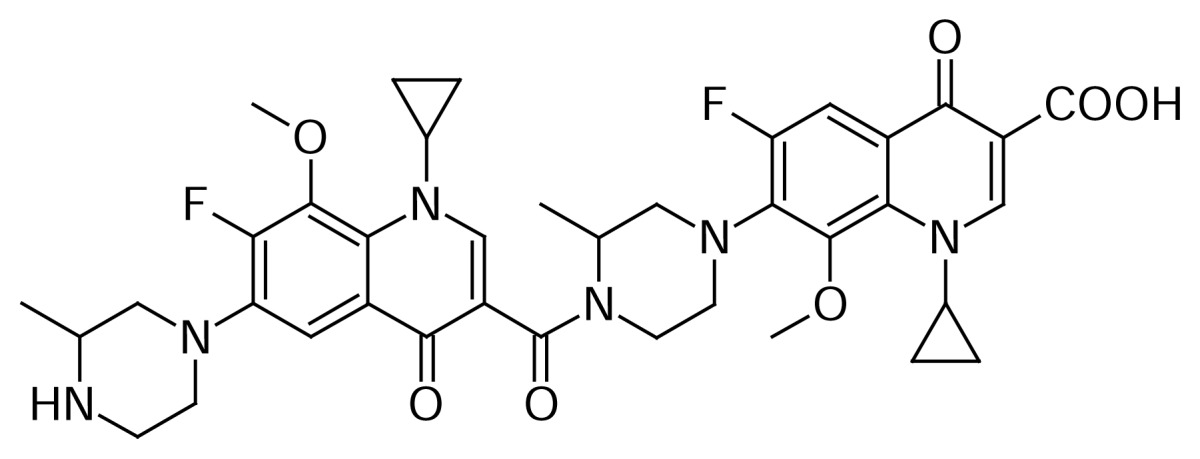 **11**	Self Condensation of **1**	Scheme 4
Despropylene Gatifloxacin 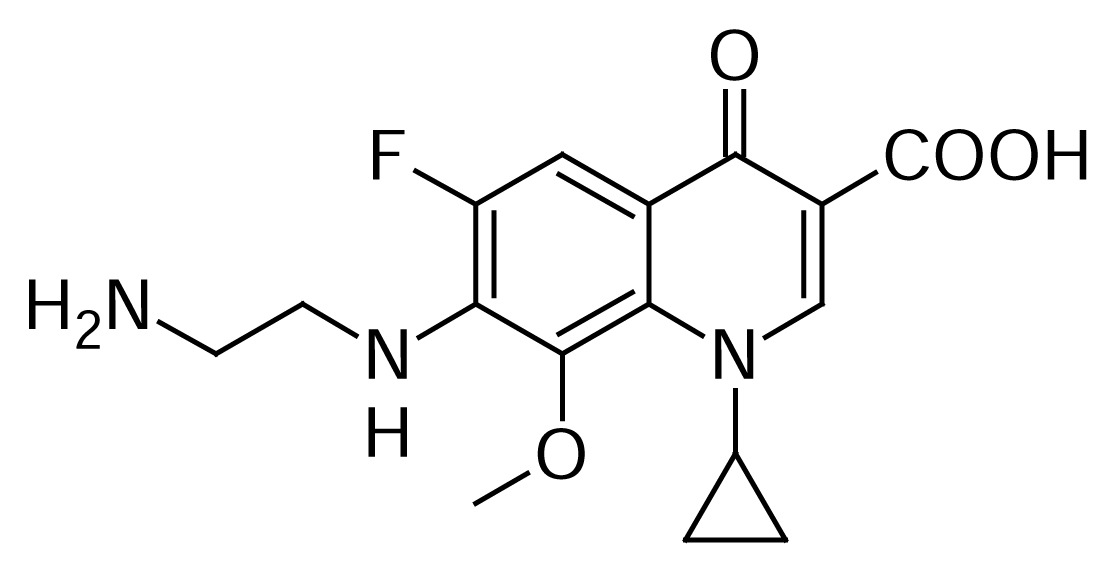 **14**	Presence of **12** in 2-methylpiperazine	Scheme 5
Gatifloxacin dimer-4 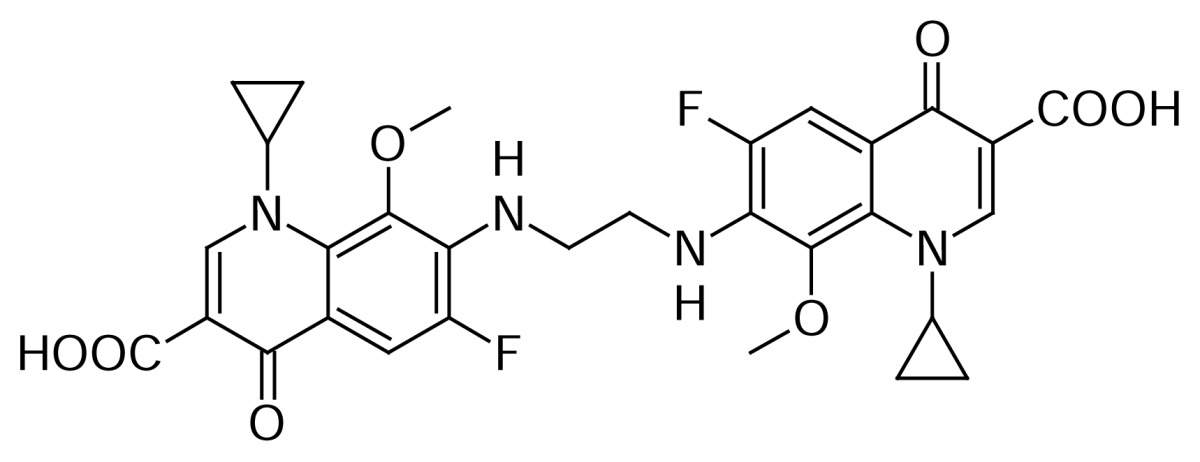 **16**	Presence of **12** in 2-methylpiperazine	Scheme 6
