# Supplementary material for: Synthesis and Characterization of Potential Dimers of Gatifloxacin – an Antibacterial Drug
Source: Sci Pharm. 2013 Feb 18;81(3):651–62. doi: 10.3797/scipharm.1212-21 (PMC3791930; doi:10.3797/scipharm.1212-21)
Supplement: Supplementary file 1 — Supporting information containing 1H-NMR spectra (6, 9, 11, 14, 16), 13C-NMR spectra (9, 11, 14), HRMS & elemental analyses (6, 9, 11, 14, 16), IR spectra (6, 9, 11, 14, 16), HPLC purity spectra (6, 9, 11, 14, 16), and the impurity mixture chromatogram of dimers are available in the online version (Format: PDF, Size: ca. 1.0 MB): http://dx.doi.org/10.3797/scipharm.1212-21. [file scipharm-2013-81-651-supp.pdf]

Scientia Pharmaceutica www.scipharm.at

Open Access

Supporting Information to

Synthesis and Characterization of Potential Dimers of Gatifloxacin – an Antibacterial Drug

Srinivas GARAGA, Ambati V. RAGHAVA REDDY, Koilpillai Joseph PRABAHAR, Raghu Babu KORUPOLU, Paul Douglas SANASI

Published in Sci Pharm. 2013; 81: 651–662 doi:10.3797/scipharm.1212-21

Available from: http://dx.doi.org/10.3797/scipharm.1212-21

© Garaga *et al.*; licensee Österreichische Apotheker-Verlagsgesellschaft m. b. H., Vienna, Austria.

This is an Open Access article distributed under the terms of the Creative Commons Attribution License (http://creativecommons.org/licenses/by/3.0/), which permits unrestricted use, distribution, and reproduction in any medium, provided the original work is properly cited.

Table of Contents

^{1}H-NMR of Compound **6**
HRMS & Elemental analysis of Compound **6**
IR Spectrum of Compound **6**
HPLC purity of Compound **6**
^{1}H-NMR of Compound **9**
^{13}C-NMR of Compound **9**
HRMS & Elemental analysis of Compound **9**
IR Spectrum of Compound **9**
HPLC Purity of Compound **9**
^{1}H-NMR of Compound **11**
^{13}C-NMR of Compound **11**
HRMS & Elemental analysis of Compound **11**
IR Spectrum of Compound **11**
HPLC Purity of Compound **11**
^{1}H-NMR of Compound **14**
^{13}C-NMR of Compound **14**
HRMS & Elemental analysis of Compound **14**
IR Spectrum of Compound **14**
HPLC Purity of Compound **14**
^{1}H-NMR of Compound **16**
HRMS & Elemental analysis of Compound **16**
IR Spectrum of Compound **16**
HPLC Purity of Compound **16**
Impurity mixture of dimers

^{1}H-NMR of Compound 6

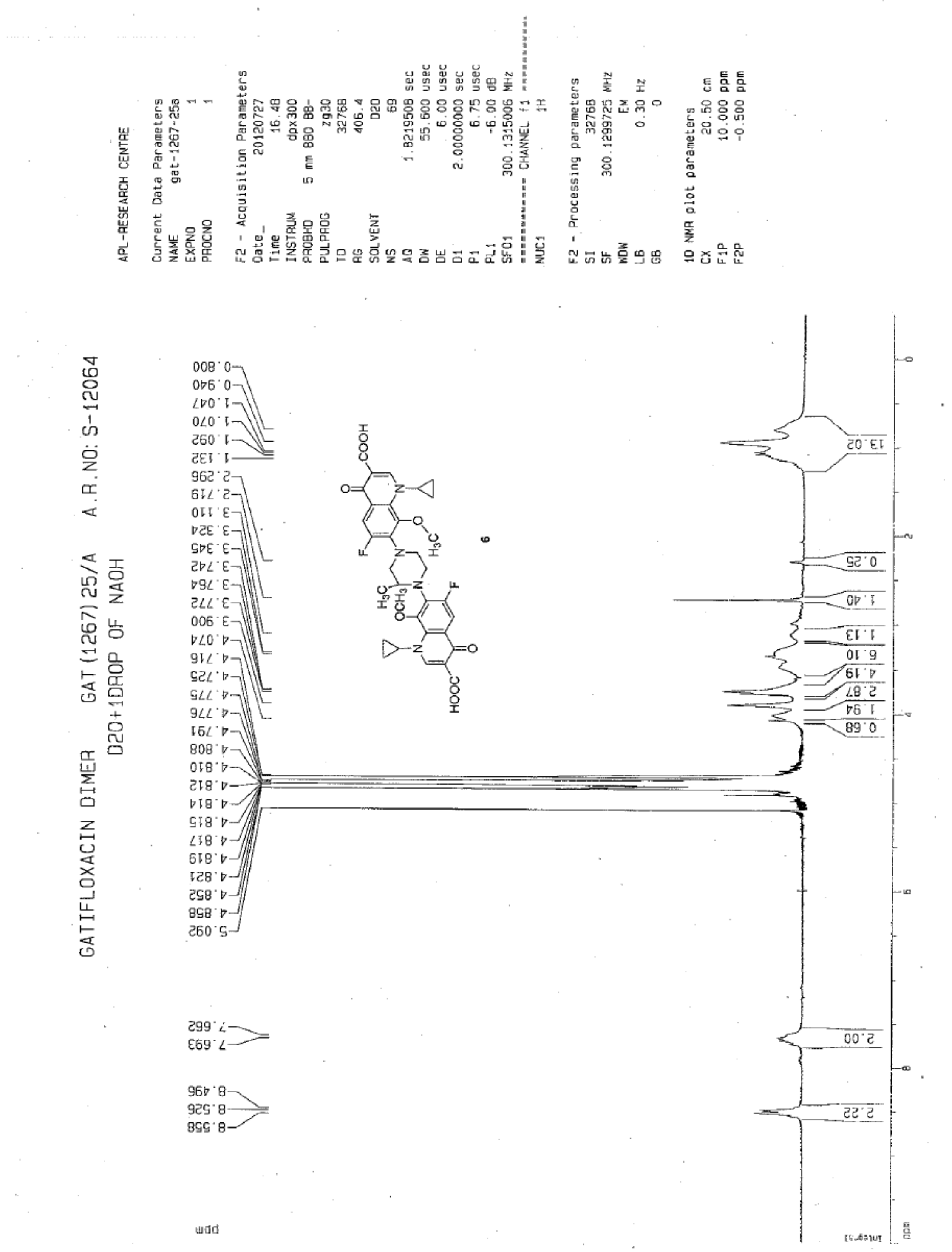

HRMS & Elemental analysis of Compound 6

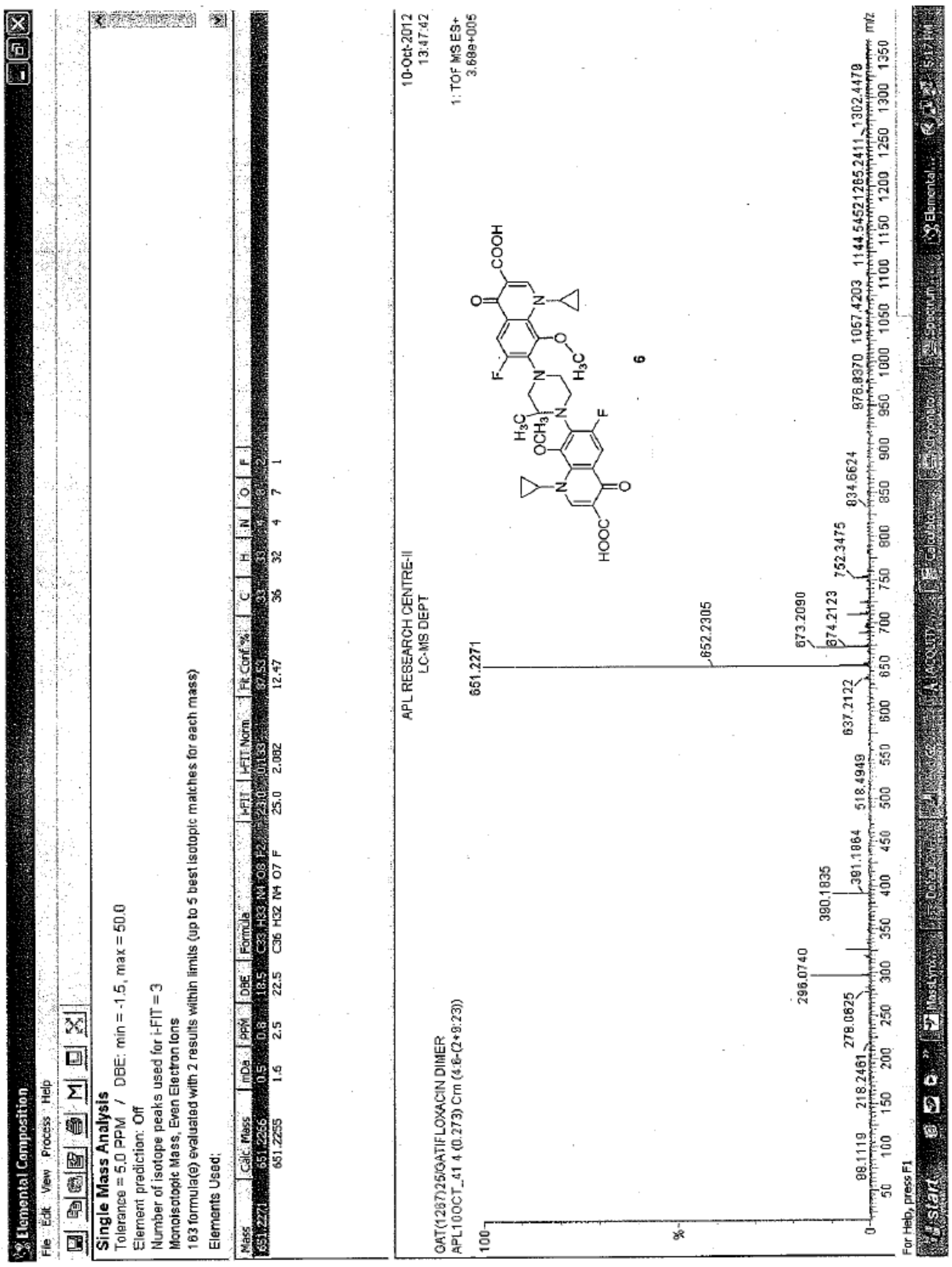

IR Spectrum of Compound 6

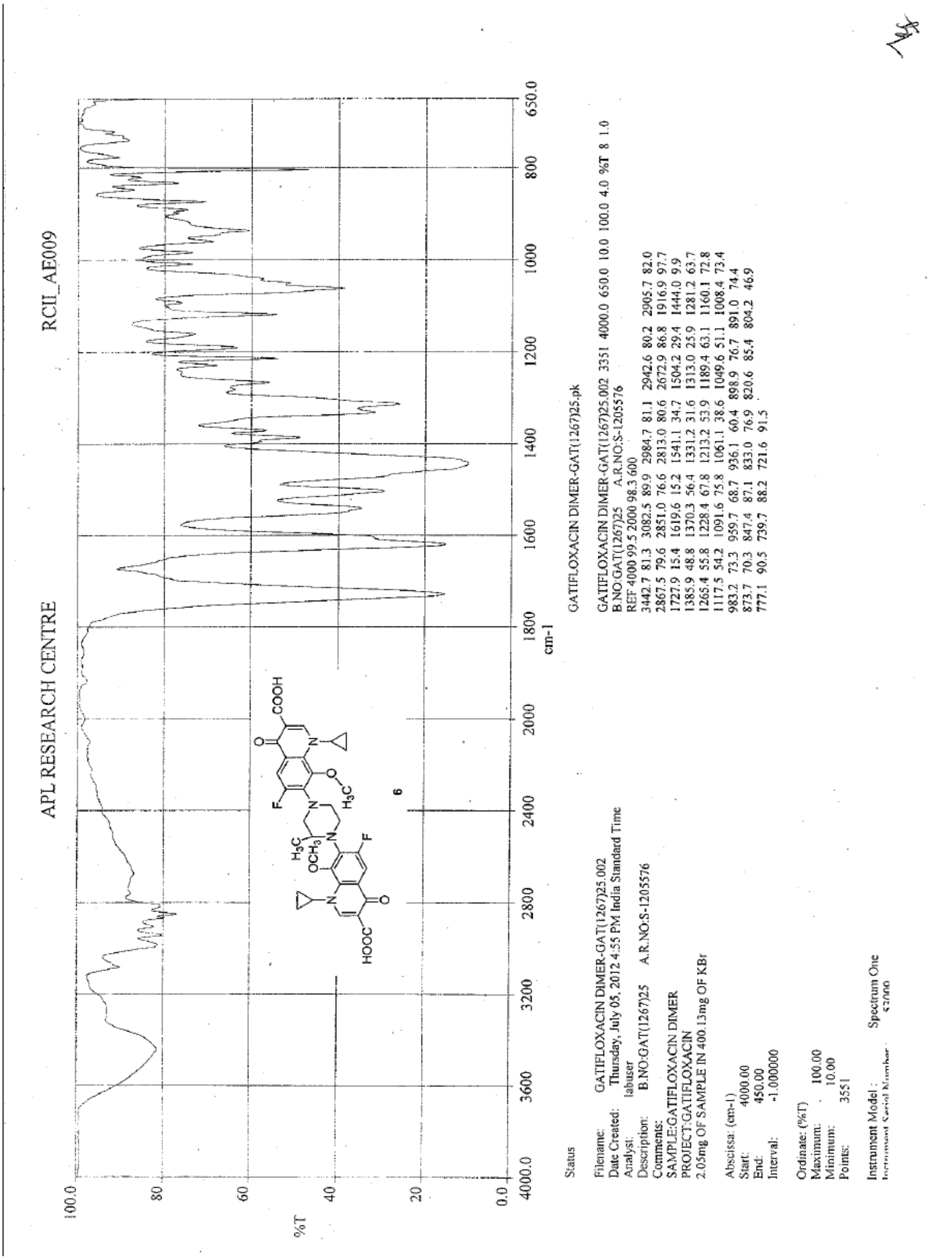

HPLC purity of Compound 6

APL RESEARCH CENTRE
ANALYTICAL RESEARCH DEPARTMENT

INSTRUMENT ID : RCII_AE136 PROJECT NAME : SEP_2012\RCII_AE136

Sample ID	:	GAT(1267)25/Gatifloxacin Dimer(M/Z=650)	Proc. Chnl. Descr :	PDA 293.0 nm
			Date Acquired :	9/18/2012 11:06:53 AM IST
Run Time	:	72.0 Minutes	Acq. Method Set :	Gatifloxacin_RS
Vial	:	8		
Injection	:	1	Date Processed :	9/18/2012 12:23:02 PM IST
Injection Volume :		20.00 ul	Processing Method :	Gatifloxacin_RS_Pro

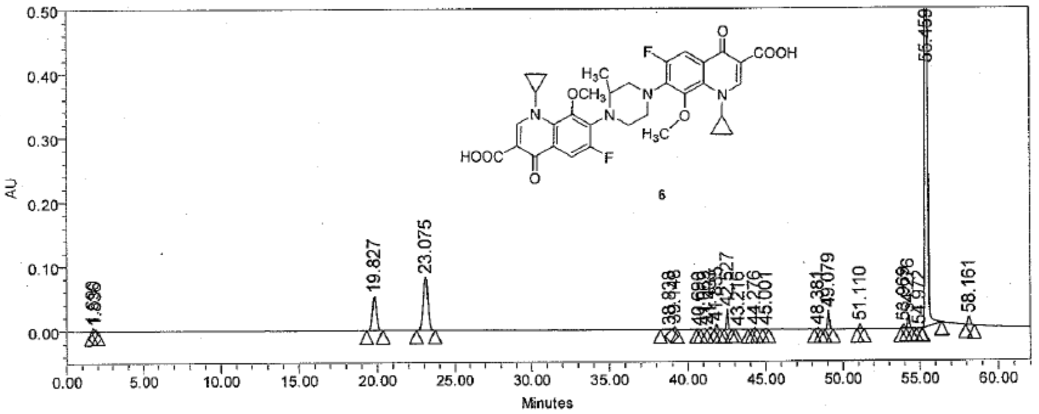

Peak Results

	RT (min)	Area (µV*sec)	% Area	Name
1	1.69	47161	0.21	Peak24
2	1.84	22212	0.10	Peak25
3	19.83	988357	4.49	Peak26
4	23.07	1773743	8.05	Peak27
5	38.84	30405	0.14	Peak28
6	39.15	38609	0.18	Peak29
7	40.69	2126	0.01	Peak30
8	41.07	9778	0.04	Peak31
9	41.43	23563	0.11	Peak32
10	41.83	54284	0.25	Peak33
11	42.53	237251	1.08	Peak34
12	43.22	5236	0.02	Peak35
13	44.28	13769	0.06	Peak36
14	45.00	13153	0.06	Peak37
15	48.38	15729	0.07	Peak38
16	49.08	239113	1.09	Peak39

	RT (min)	Area (µV*sec)	% Area	Name
17	51.11	65212	0.30	Peak40
18	53.97	48154	0.22	Peak41
19	54.28	155592	0.71	Peak42
20	54.97	5371	0.02	Peak43
21	55.46	18107416	82.19	Peak44
22	58.16	135755	0.62	Peak45

^{1}H-NMR of Compound 9

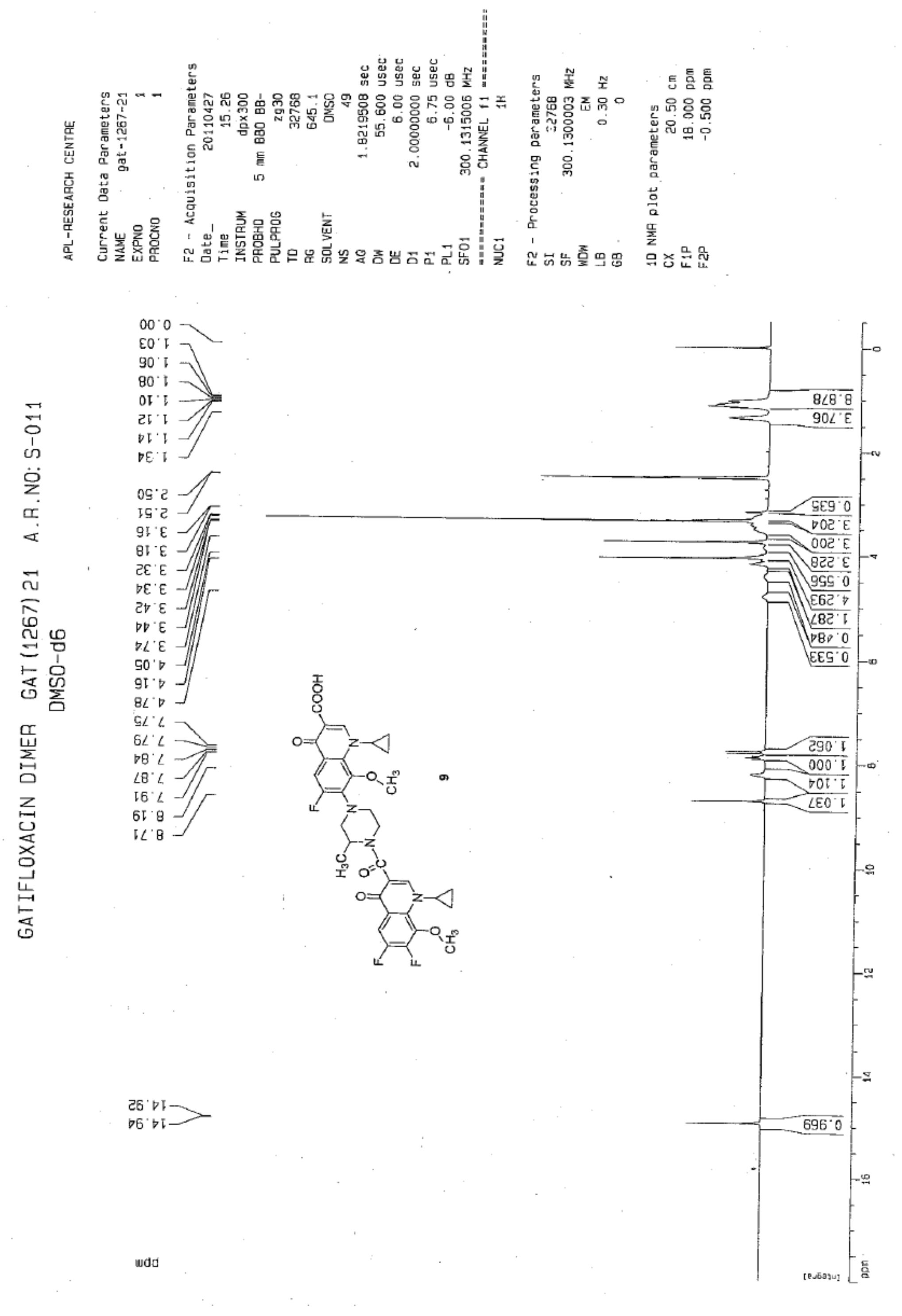

^{13}C-NMR of Compound 9

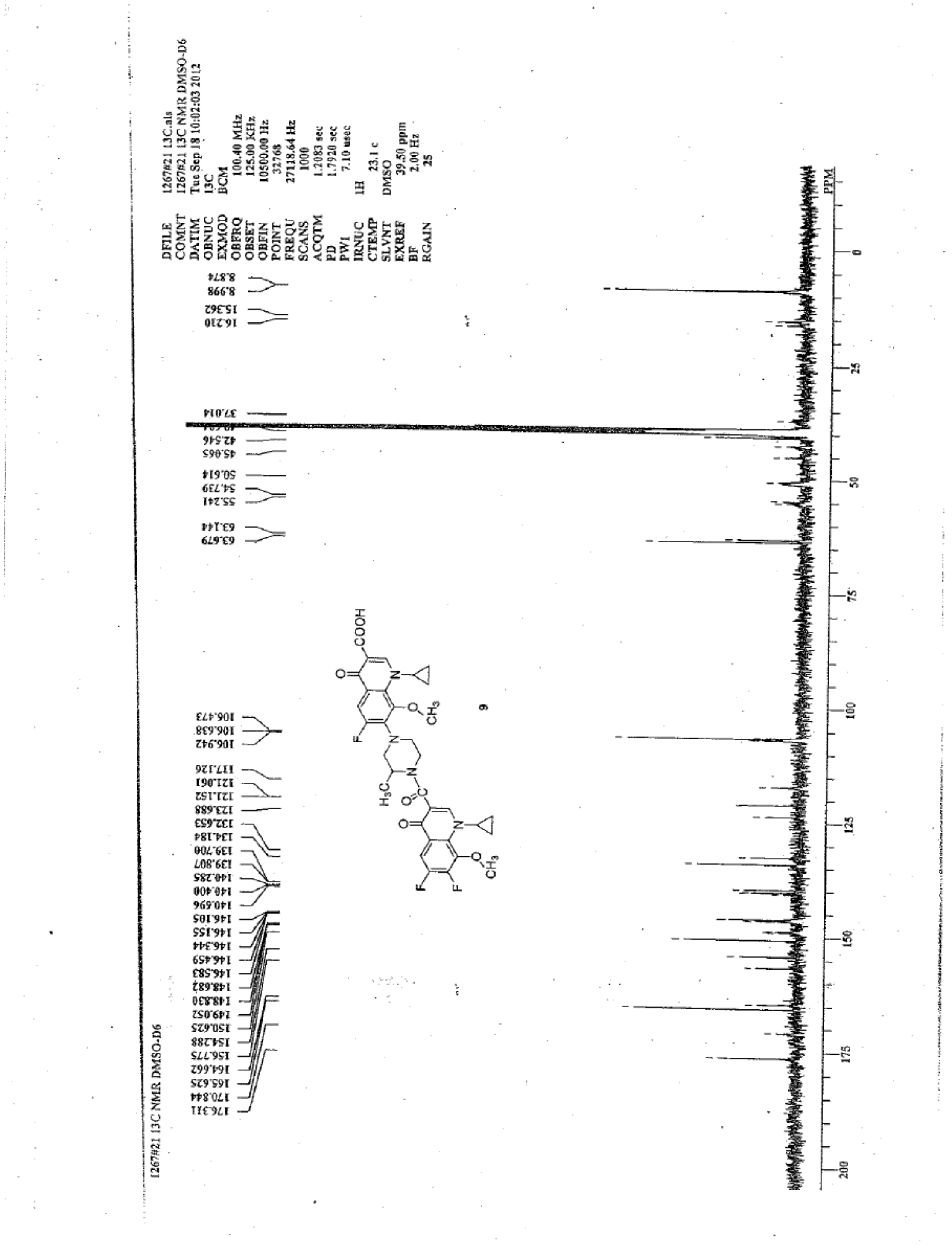

HRMS & Elemental analysis of Compound 9

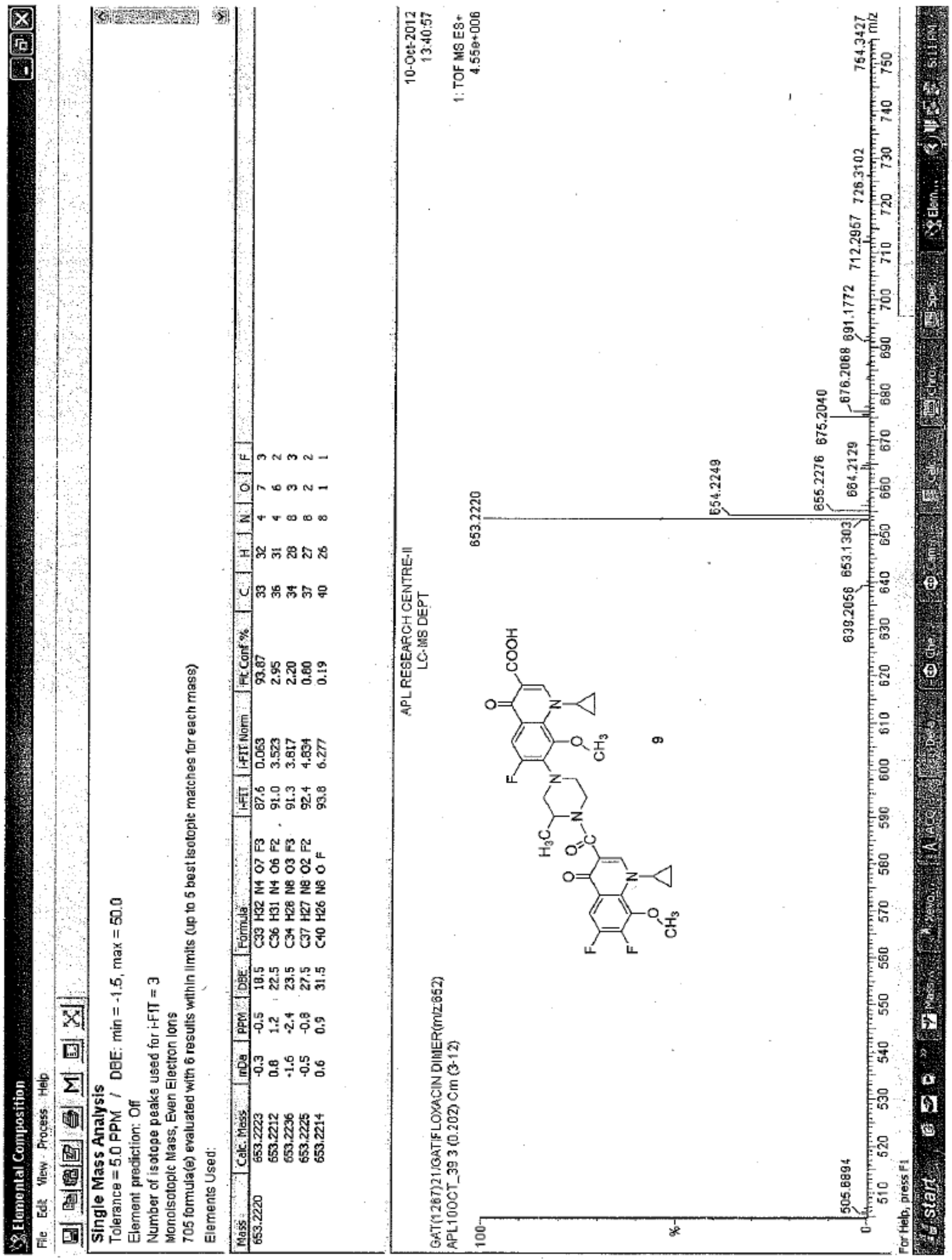

IR Spectrum of Compound 9

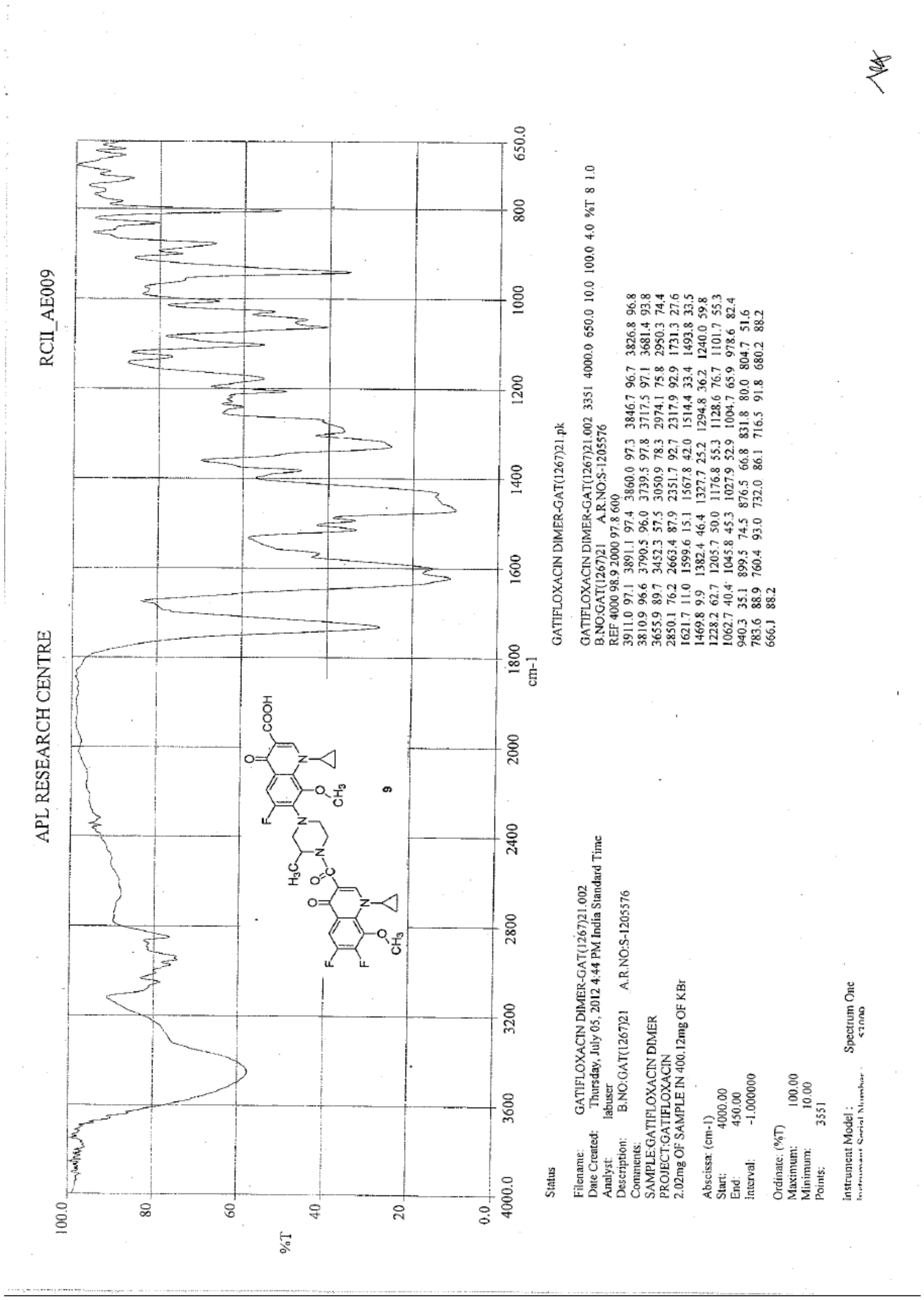

HPLC Purity of Compound 9

APL RESEARCH CENTRE
ANALYTICAL RESEARCH DEPARTMENT

INSTRUMENT ID : RCII_AE136 PROJECT NAME : SEP_2012\RCII_AE136

Sample ID :	GAT(1267)21/Gatifloxacin Dimer(M/Z=652)	Proc. Chnl. Descr :	PDA 293.0 nm
		Date Acquired :	17/9/2012 19:01:30 IST
Run Time :	72.0 Minutes	Acq. Method Set :	Gatifloxacin_RS
Vial :	3		
Injection :	1	Date Processed :	18/9/2012 10:12:37 IST
Injection Volume :	20.00 ul	Processing Method :	Gatifloxacin_RS_Pro

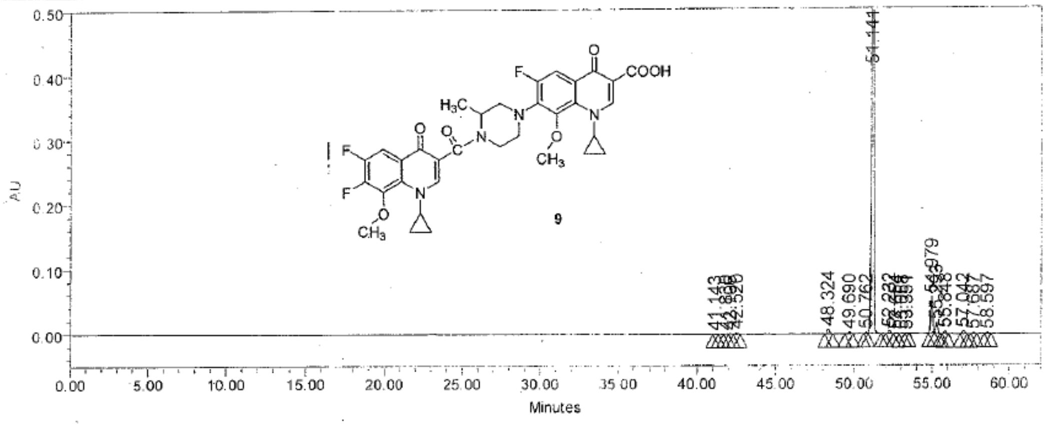

Peak Results

	RT (min)	Area (µV*sec)	% Area	Name
1	41.14	3860	0.04	Peak1
2	41.83	5788	0.05	Peak39
3	42.11	5346	0.05	Peak3
4	42.52	20900	0.19	Peak4
5	48.32	70927	0.65	Peak5
6	49.69	25733	0.23	Peak6
7	50.76	17748	0.16	Peak7
8	51.14	10084051	91.71	Peak8
9	52.23	37709	0.34	Peak9
10	52.65	6291	0.06	Peak10
11	53.06	3914	0.04	
12	53.35	3923	0.04	Peak11
13	54.98	488085	4.44	Peak12
14	55.29	120896	1.10	Peak13
15	55.85	31073	0.28	Peak14
16	57.04	35823	0.33	Peak15
17	57.69	12467	0.11	Peak16
18	58.60	20639	0.19	Peak17

^{1}H-NMR of Compound 11

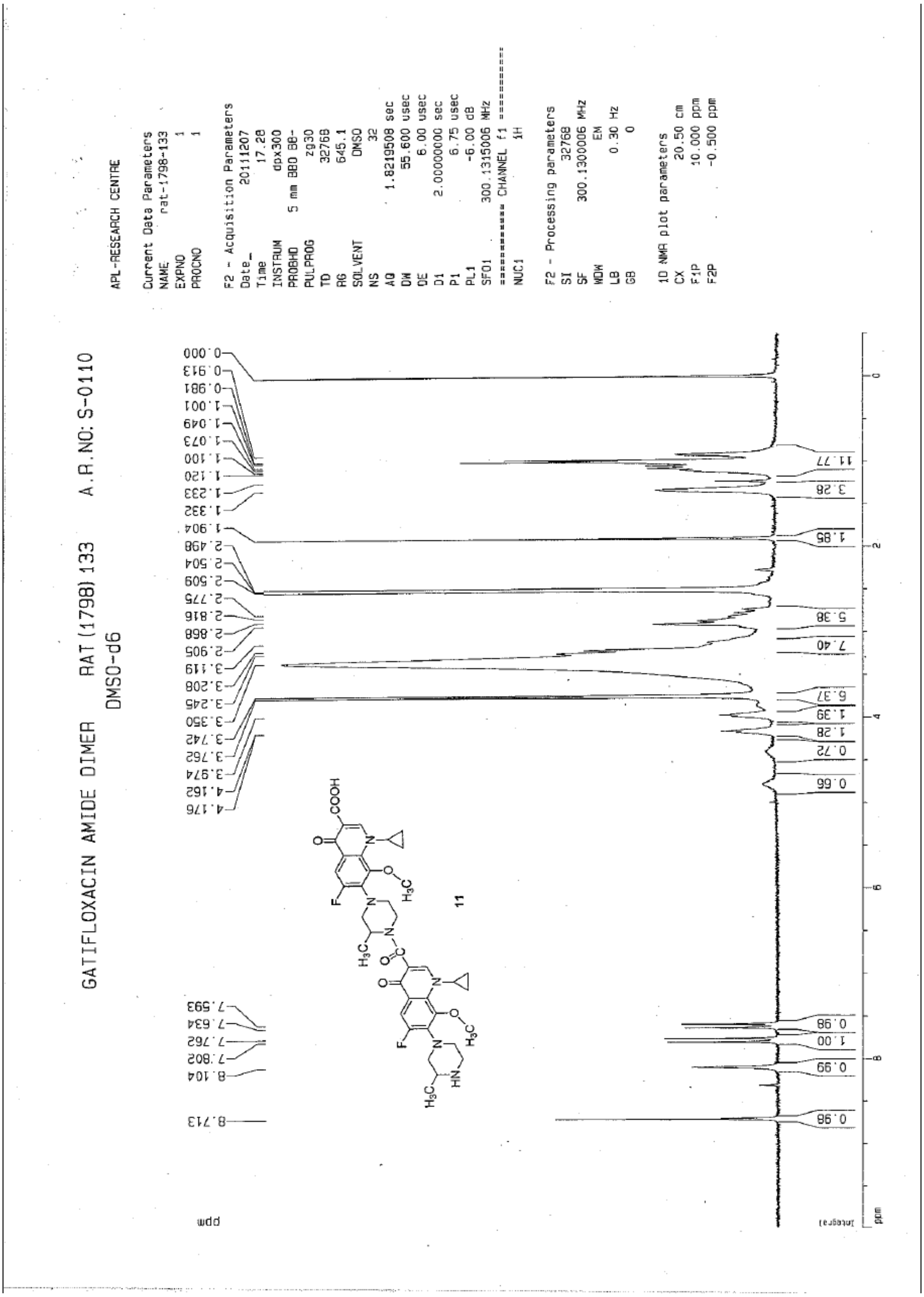

^{13}C-NMR of Compound 11

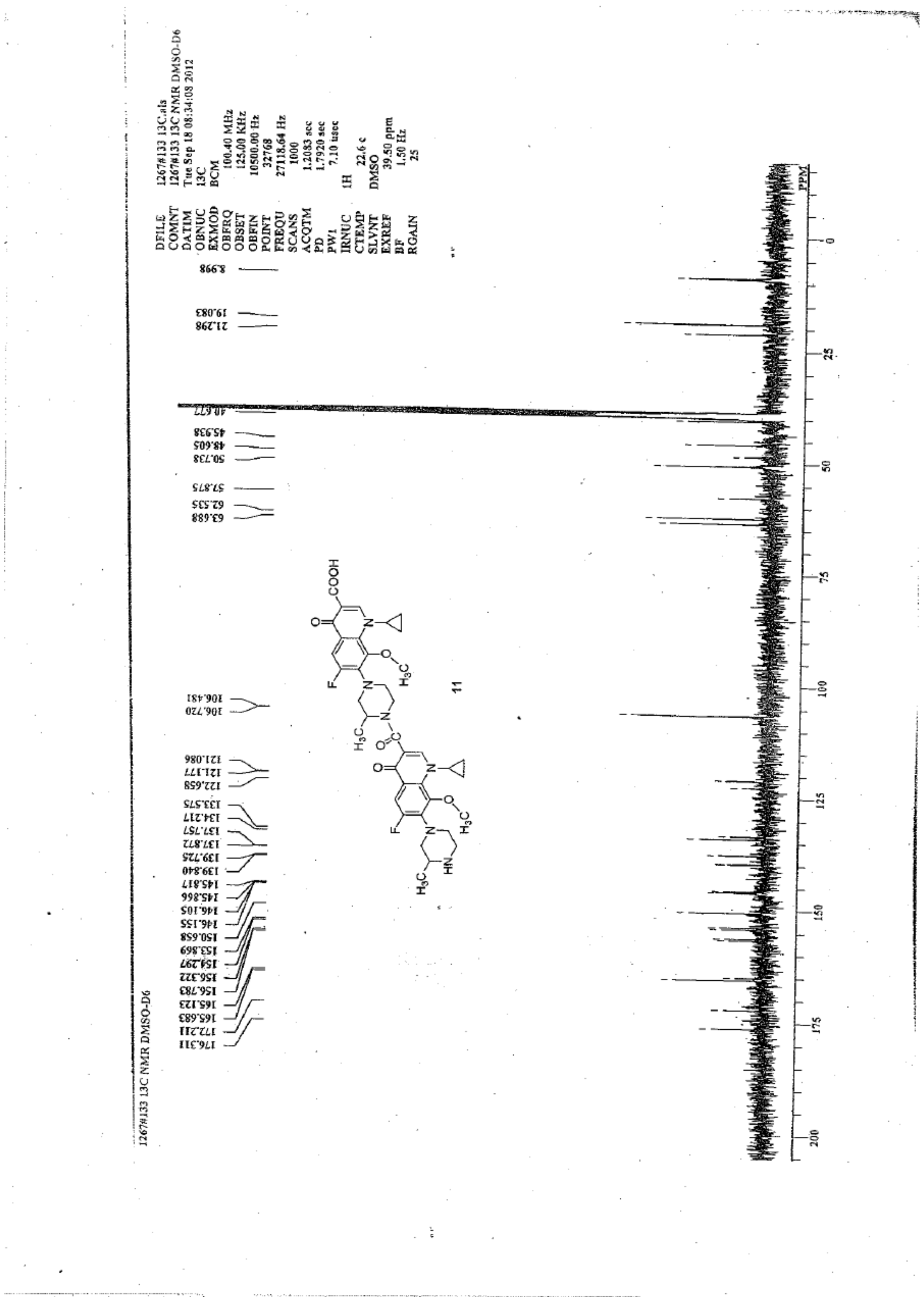

HRMS & Elemental analysis of Compound 11

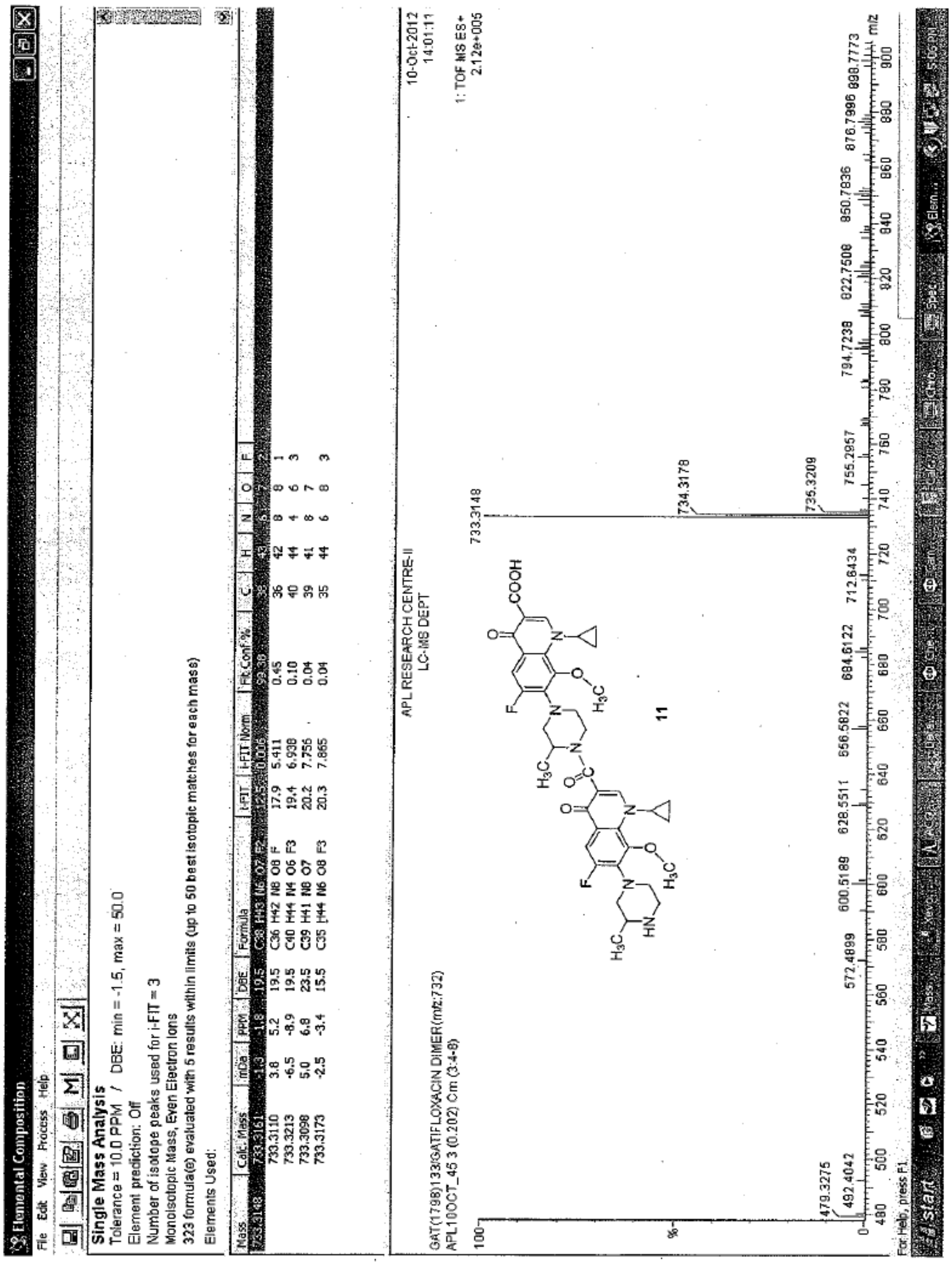

IR Spectrum of Compound 11

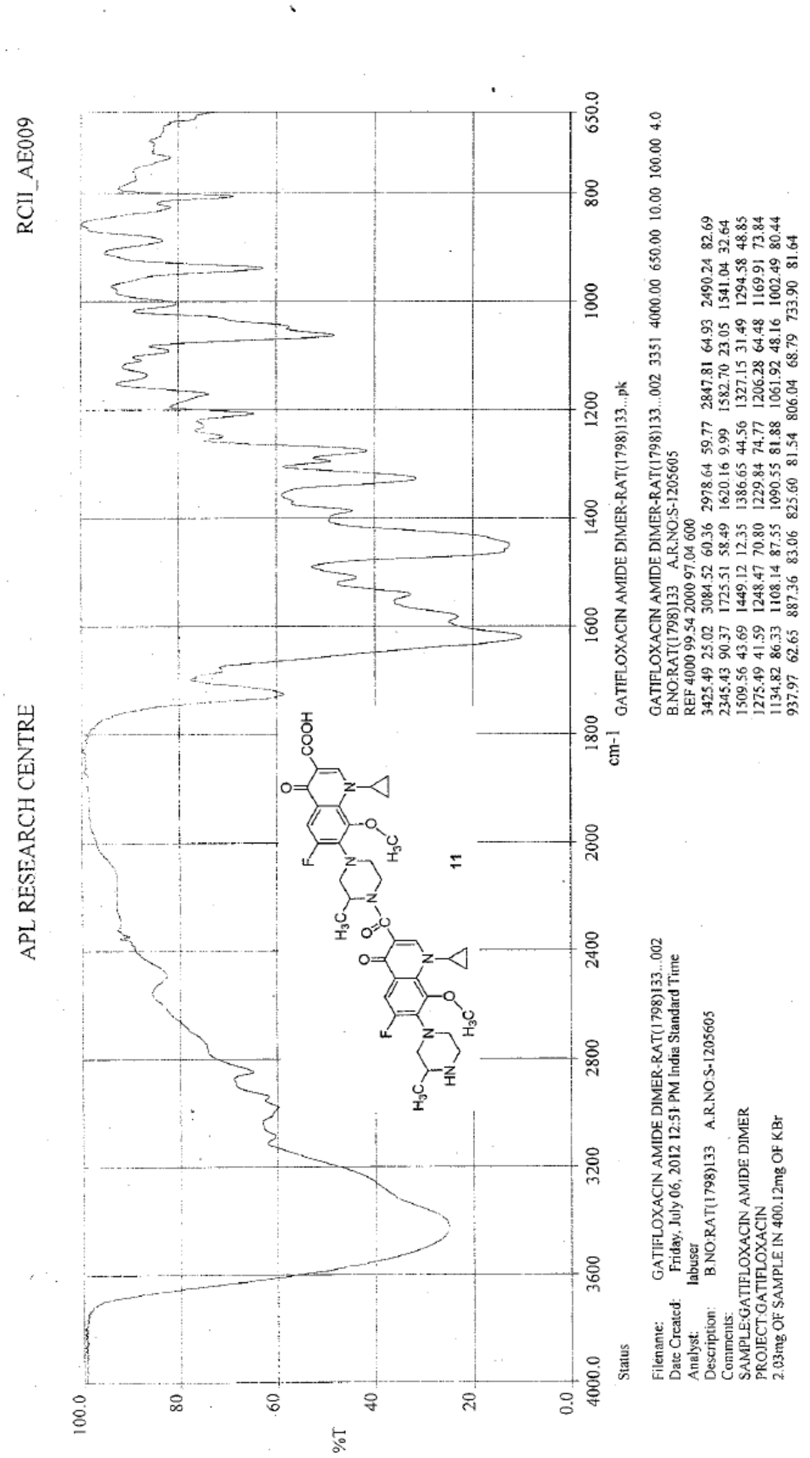

HPLC Purity of Compound 11

APL RESEARCH CENTRE
ANALYTICAL RESEARCH DEPARTMENT

INSTRUMENT ID : RCII_AE136 PROJECT NAME : SEP_2012\RCII_AE136

Sample ID	:	GAT(1798)133/Gatifloxacin Dimer(M/Z=732)	Proc. Chnl. Descr	:	PDA 293.0 nm
Run Time	:	72.0 Minutes	Date Acquired	:	17/9/2012 17:48:13 IST
Vial	:	5	Acq. Method Set	:	Gatifloxacin_RS
Injection	:	1	Date Processed	:	18/9/2012 9:51:28 IST
Injection Volume	:	20.00 ul	Processing Method	:	Gatifloxacin_RS_Pro

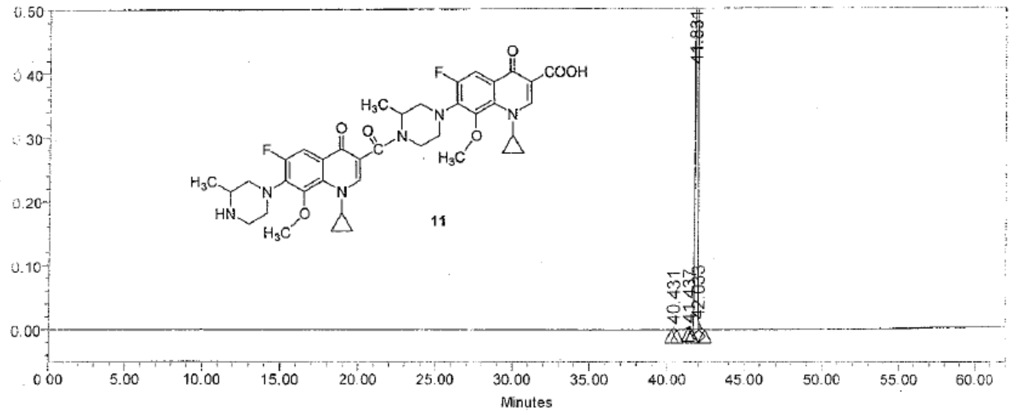

Peak Results

	RT (min)	Area (µV*sec)	% Area	Name
1	40.43	25985	0.12	Peak38
2	41.44	18527	0.09	Peak1
3	41.83	21379175	99.42	Peak39
4	42.03	80715	0.38	Peak40

^{1}H-NMR of Compound 14

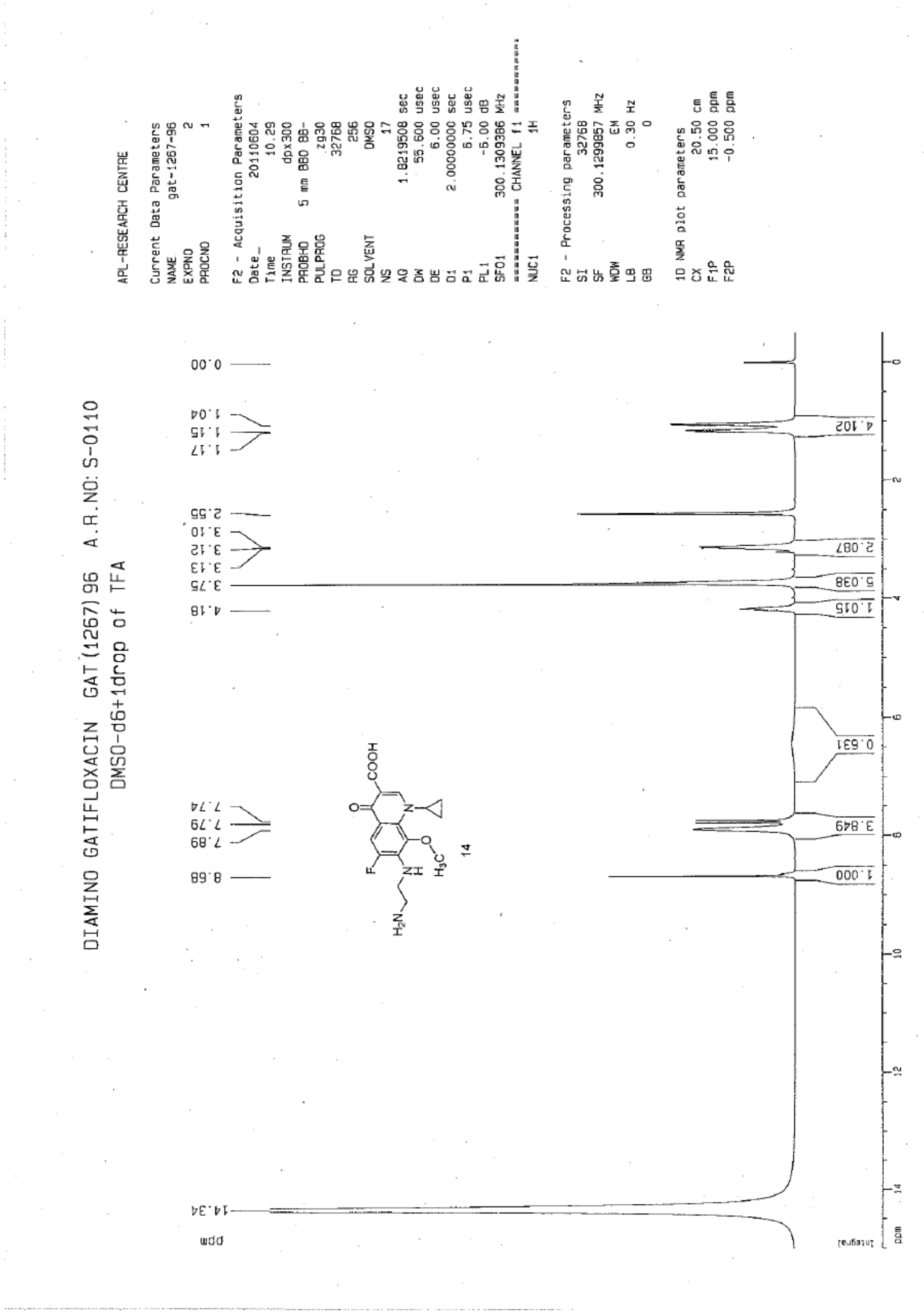

^{13}C-NMR of Compound 14

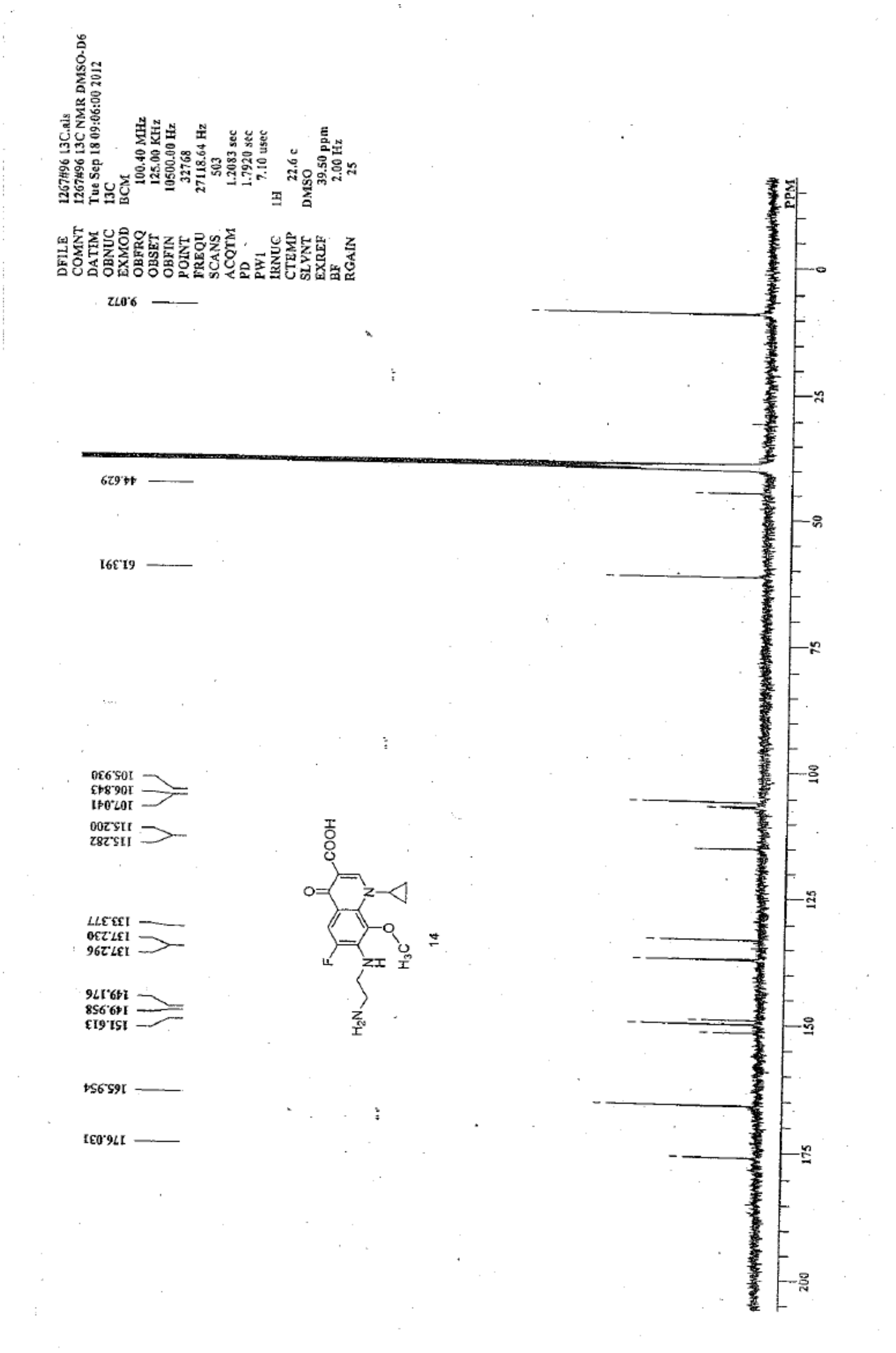

HRMS & Elemental analysis of Compound 14

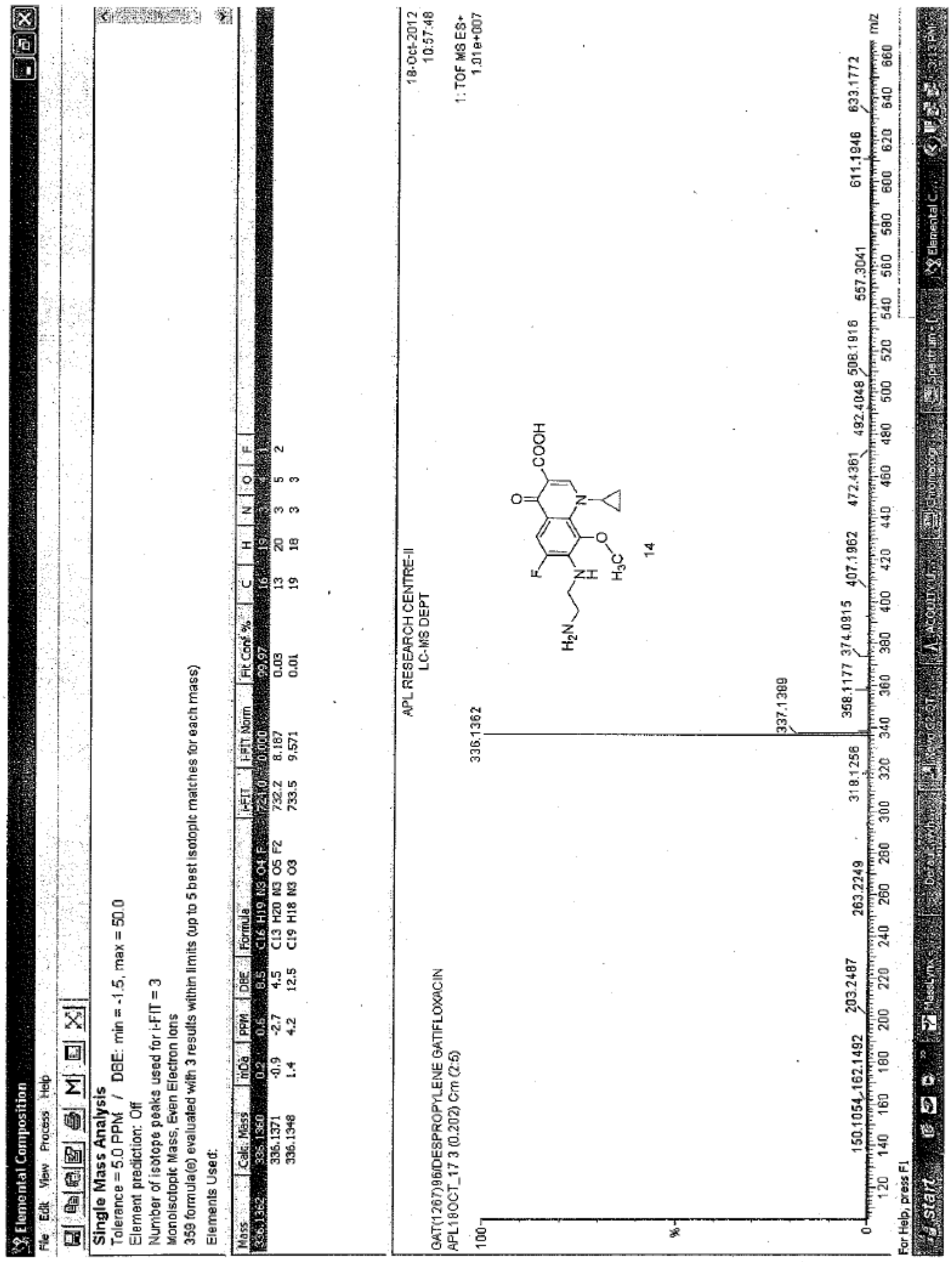

IR Spectrum of Compound 14

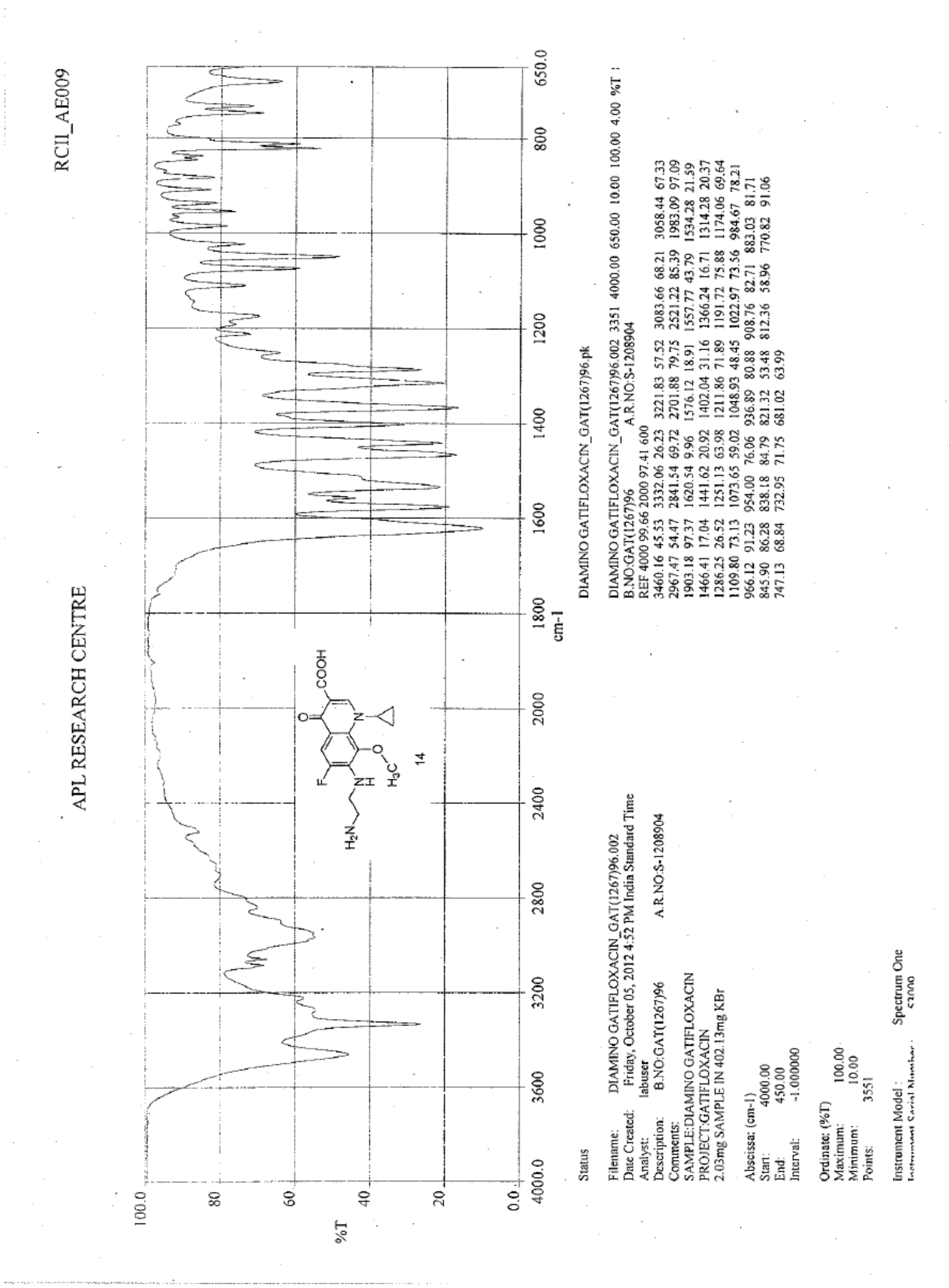

HPLC Purity of Compound 14

APL RESEARCH CENTRE
ANALYTICAL RESEARCH DEPARTMENT

INSTRUMENT ID : ARE_522_PDA PROJECT NAME : JUN_2011\ARE_522

Sample ID :	GAT(1267)96/ DiaminoGatifloxacin	Proc. Chnl. Descr :	PDA 293.0 nm
Run Time :	55.0 Minutes	Date Acquired :	6/3/2011 4:29:13 PM IST
Vial :	29	Acq. Method Set :	Gatifloxacin_RS_METH
Injection :	1	Date Processed :	6/4/2011 9:28:15 AM IST
Injection Volume :	20.00 ul	Processing Method :	Gatifloxacin_ RS_PROC

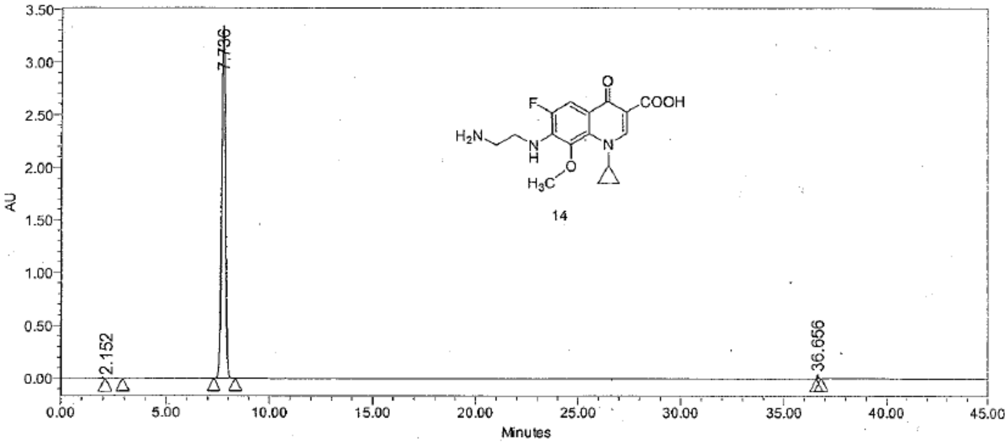

Peak Results

	RT (min)	Area (µV*sec)	%Area	Name
1	2.15	24031	0.05	Peak1
2	7.74	45299002	99.64	Peak2
3	36.66	139549	0.31	Peak3

^{1}H-NMR of Compound 16

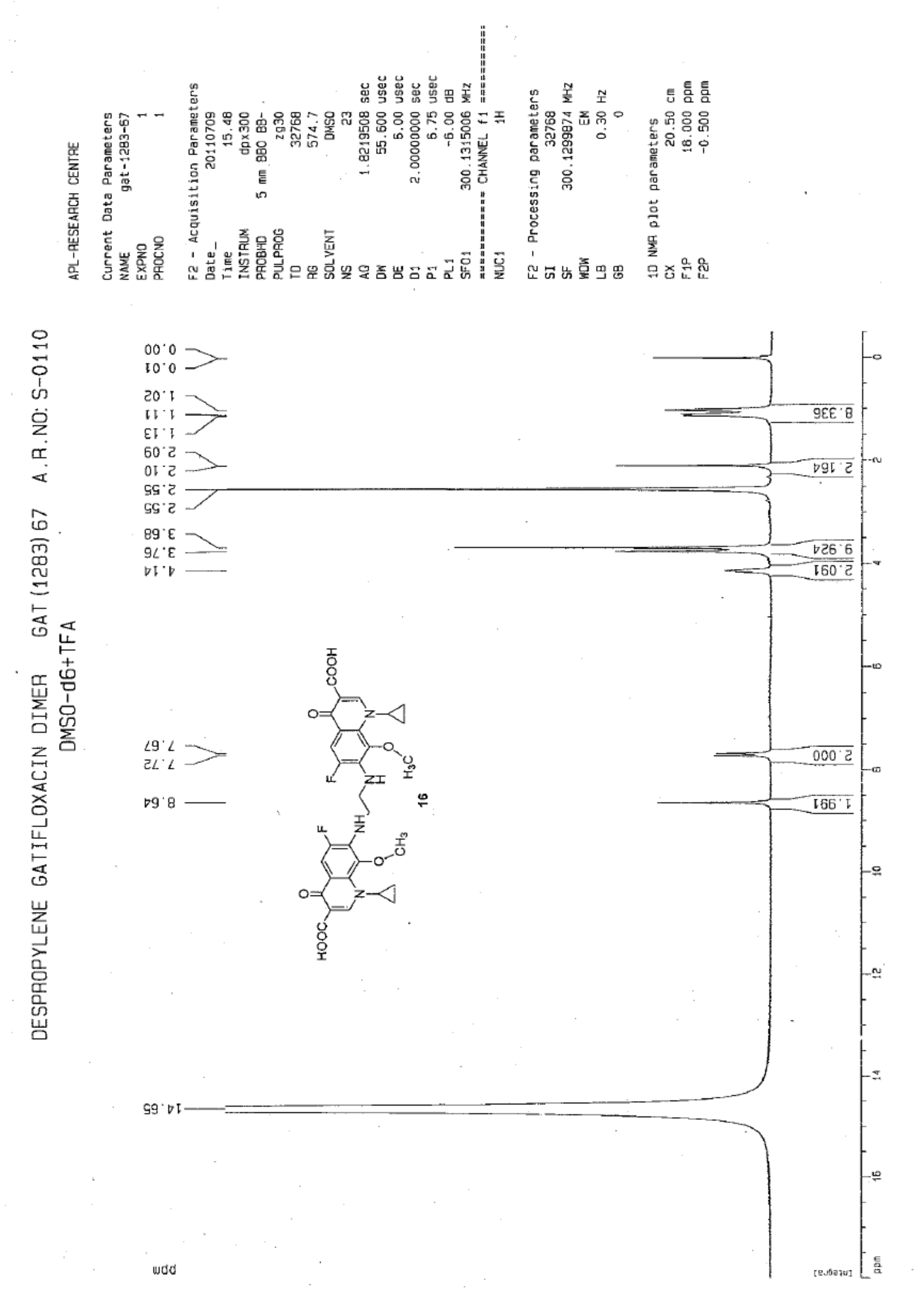

HRMS & Elemental analysis of Compound 16

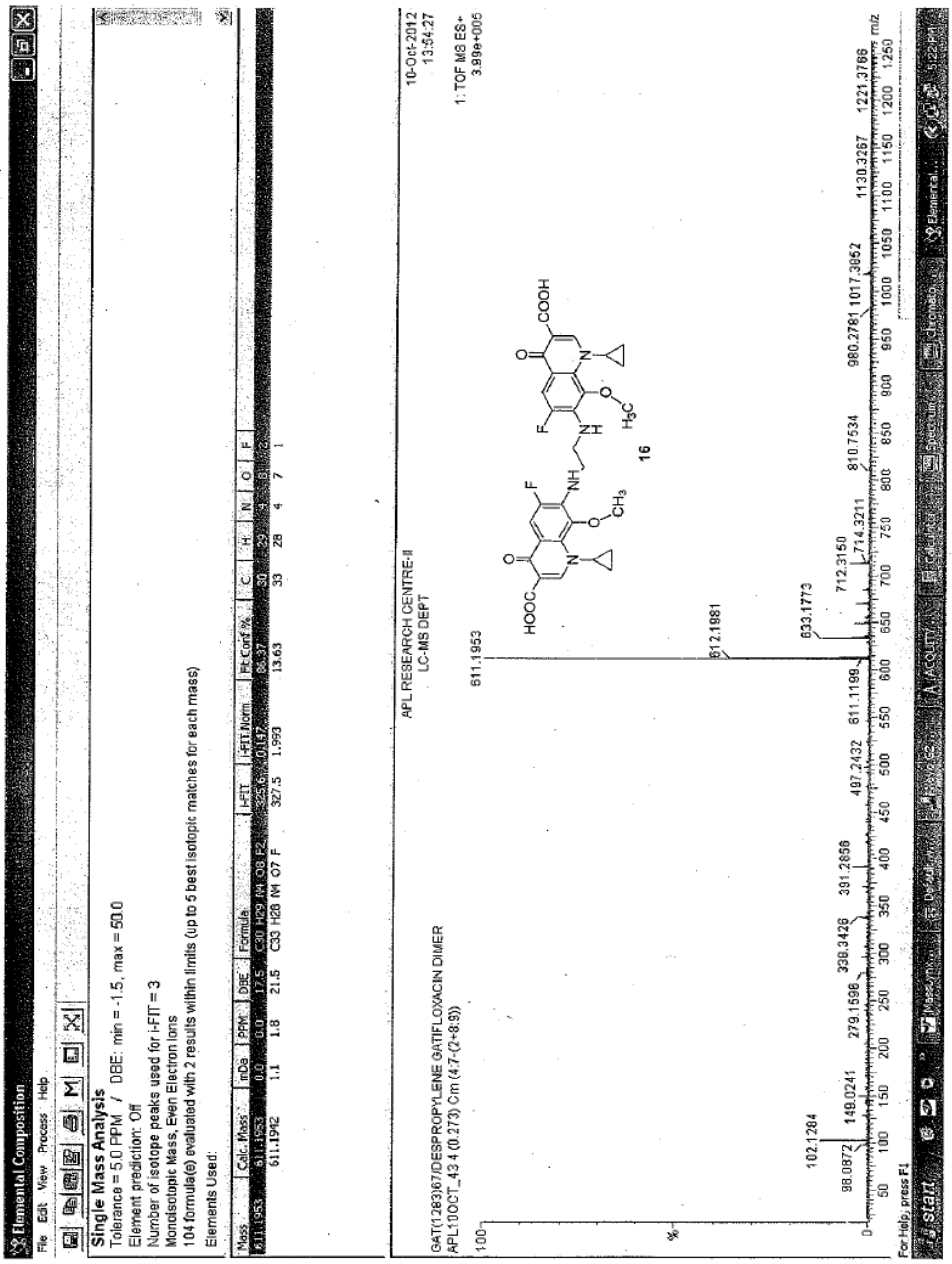

IR Spectrum of Compound 16

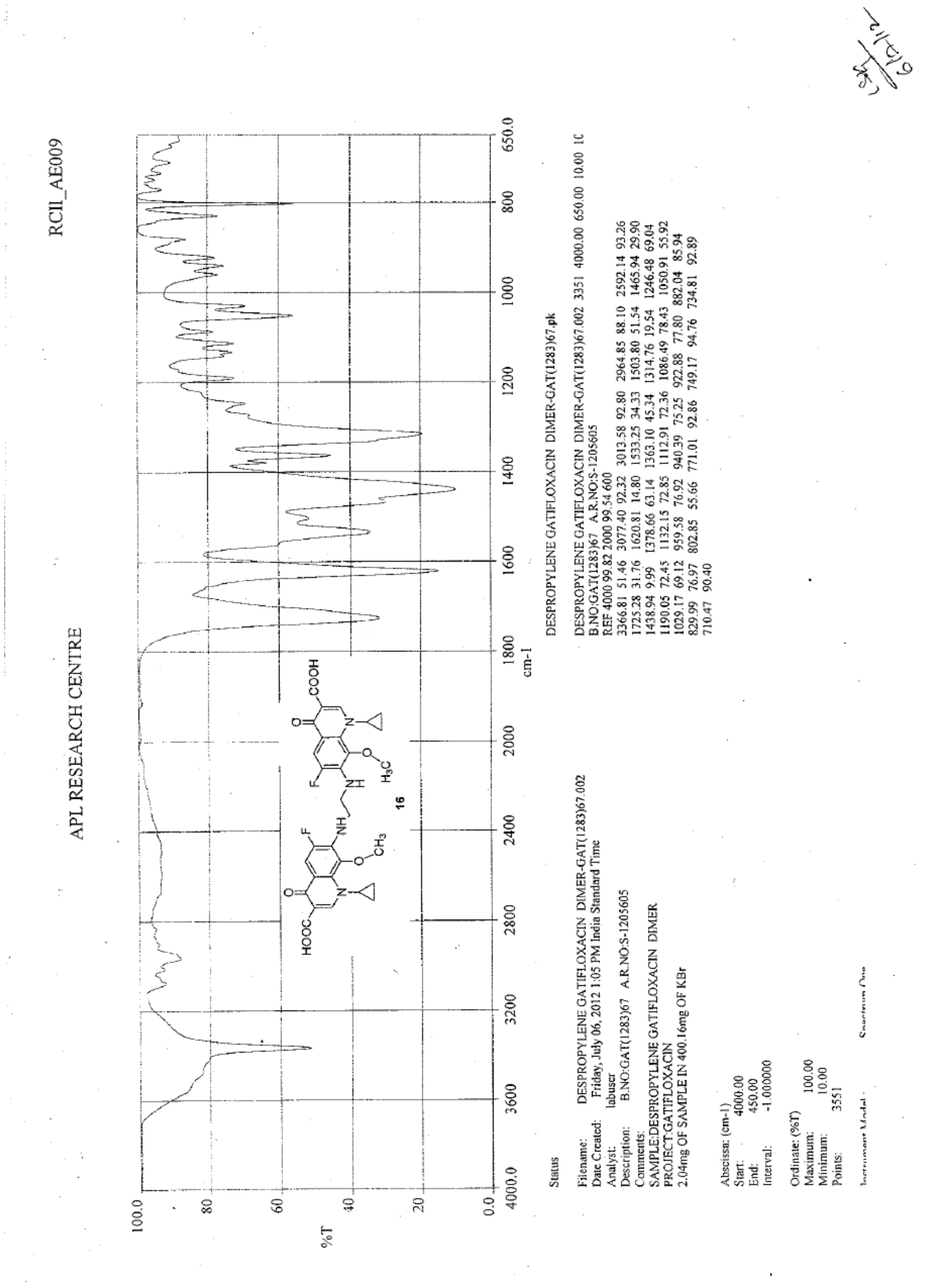

HPLC Purity of Compound 16

APL RESEARCH CENTRE
ANALYTICAL RESEARCH DEPARTMENT

INSTRUMENT ID : RCII_AE136 PROJECT NAME : SEP_2012\RCII_AE136

Sample ID :	GAT(1283)67/Gatifloxacin Dimer(M/Z=610)	Proc. Chnl. Descr :	PDA 293.0 nm
Run Time :	72.0 Minutes	Date Acquired :	17/9/2012 16:34:56 IST
Vial :	4	Acq. Method Set :	Gatifloxacin_RS
Injection :	1	Date Processed :	18/9/2012 10:11:24 IST
Injection Volume :	20.00 ul	Processing Method :	Gatifloxacin_RS_Pro

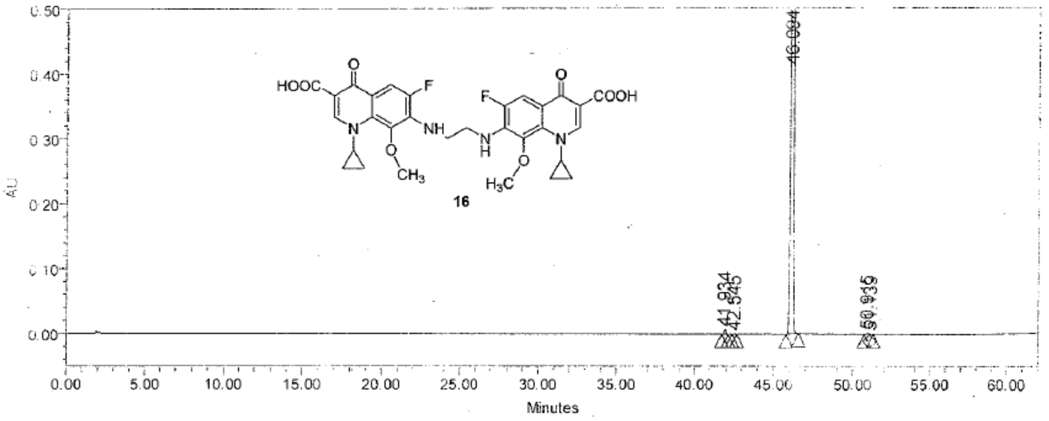

Peak Results

	RT (min)	Area (µV*sec)	% Area	Name
1	41.93	46546	0.30	Peak2
2	42.55	7278	0.05	Peak4
3	46.09	15378098	99.45	Peak5
4	50.92	22885	0.15	Peak7
5	51.14	7595	0.05	Peak8

Impurity mixture of dimers

APL RESEARCH CENTRE-II
ANALYTICAL RESEARCH DEPARTMENT

NSTRUMENT ID RCII_AE136 PROJECT NAME SEP_2012\RCII_AE136

Sample ID:	Gatifloxacin Dimers Impurtty Mix	Proc. Chnl. Descr.:	PDA 293.0 nm
Vial:	9	Date Acquired:	9/18/2012 12:34:14 PM IST
njection :	1	Acq. Method Set:	Gatifloxacin_RS
njection Volume:	20.00 ul	Date Processed:	9/18/2012 1:48:41 PM IST
Run Time:	72.0 Minutes	Processing Method:	Gatifloxacin_RS_Pro

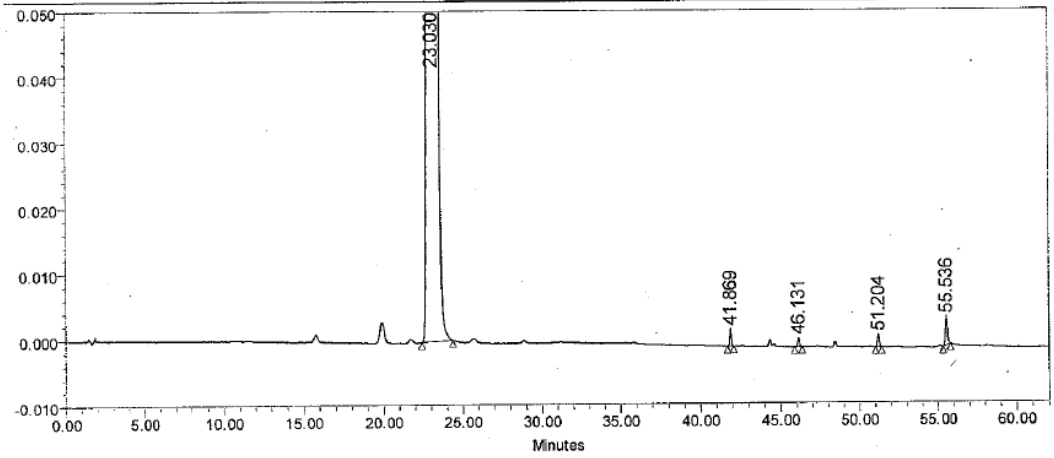

PEAK RESULTS

	RT	USP Plate Count	USP Tailing	USP Resolution	RT Ratio	Name
1	23.03	23268	1.31			Gatifloxacin
2	41.87	783805	1.03	46.66	1.82	Gatifloxacin Dimer (M/Z=732)
3	46.13	661209	0.97	20.15	2.00	Gatifloxacin Dimer (M/Z=610)
4	51.20	743010	1.00	21.51	2.22	Gatifloxacin Dimer (M/Z=652)
5	55.54	884483	1.06	17.88	2.41	Gatifloxacin Dimer (M/Z=650)
